# Synthesis and crystal structures of boryl *ortho*-silylaryl tri­fluoro­methane­sulfonates

**DOI:** 10.1107/S2056989024000264

**Published:** 2024-01-12

**Authors:** Fredrik Barnå, Matic Hribersek, Andreas Orthaber, Lukasz T. Pilarski

**Affiliations:** a Department of Chemistry-BMC, Uppsala University, Box 576, 75123, Uppsala, Sweden; b Uppsala University Ångström Laboratories, Box 523, 75120 Uppsala, Sweden; Universidad de Los Andes Mérida, Venezuela

**Keywords:** solid-state structure, aryne precursor, pinacole borane, crystal structure

## Abstract

We report the synthesis and structural characterization of three crystalline borylated *ortho*-silylaryl tri­fluoro­methane­sulfonates: 5-(4,4,5,5-tetra­methyl-1,3,2-dioxaborolan-2-yl)-2-(tri­methyl­sil­yl)phenyl tri­fluoro­methane­sulfonate, C_16_H_24_BF_3_O_5_SSi (**1a**), 4-(4,4,5,5-tetra­methyl-1,3,2-dioxaborolan-2-yl)-2-(tri­methyl­sil­yl)phenyl tri­fluoro­methane­sulfonate, C_16_H_24_BF_3_O_5_SSi (**1b**), and 2-methyl-4-(4,4,5,5-tetra­methyl-1,3,2-dioxaborolan-2-yl)-6-(tri­methyl­silyl)phen­yl tri­fluoro­methane­sulfonate, C_17_H_26_BF_3_O_5_SSi (**2**), which are versatile aryne precursors. For all three compounds, the heteroatom substituents are almost coplanar with the central aromatic moiety. C—heteroatom bonding metrics are unexceptional and fall withing the typical range of C—B, C—Si, and C—O single bonds. Despite numerous electronegative sites, only weak inter­molecular inter­actions are observed in the solid state.

## Abstract 2

The preparation and solid-state structures of three borylated *ortho*-silylaryl tri­fluoro­methane­sulfonates are reported. All compounds show the expected connectivity and unexceptional metric parameters as well as weak intra­molecular inter­actions.

## 1. Chemical context

Arynes are remarkably versatile inter­mediates in organic synthesis (Anthony *et al.*, 2021 ▸; Takikawa *et al.*, 2018 ▸; Tadross *et al.*, 2012 ▸). Their generation from *ortho*-silylaryl triflates (Shi *et al.*, 2021 ▸) using fluoride salts (Himeshima *et al.*, 1983 ▸) or other mild bases (Idiris & Jones, 2017 ▸) has enabled the development of many otherwise impossible transformations. However, *ortho*-silylaryl triflates can themselves be challenging to introduce in many chemical contexts, which has limited their usefulness. We previously showed (Demory *et al.*, 2015 ▸) that simple *ortho*-silylaryl triflate aryne precursors can be diversified in a straightforward manner by leveraging the versatility of organoboronate groups introduced *via* Ir-catalysed C—H borylation (Bisht *et al.*, 2022 ▸; Mkhalid *et al.*, 2010 ▸). Hosoya and co-workers published a closely related study showcasing a complementary reaction scope (Yoshida *et al.*, 2015 ▸). In the course of our studies, we prepared crystals of several boryl aryne precursors. 
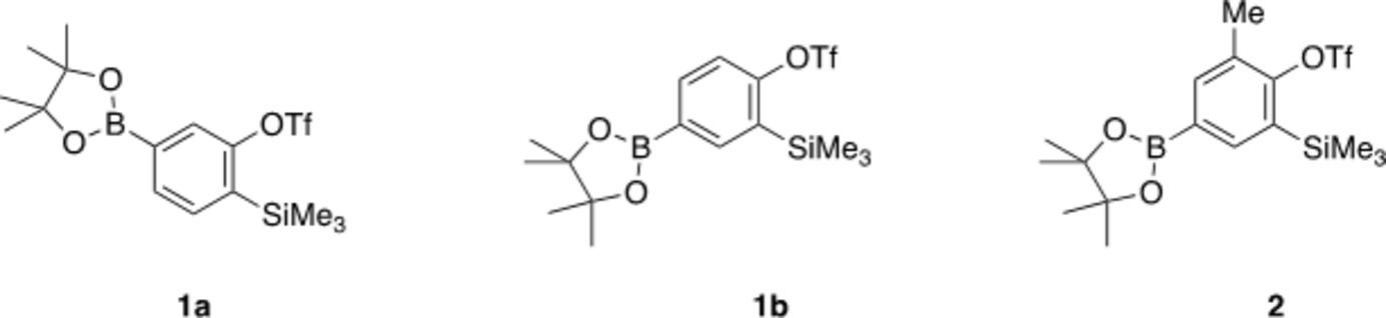


## 2. Structural commentary

**Figure 1:**
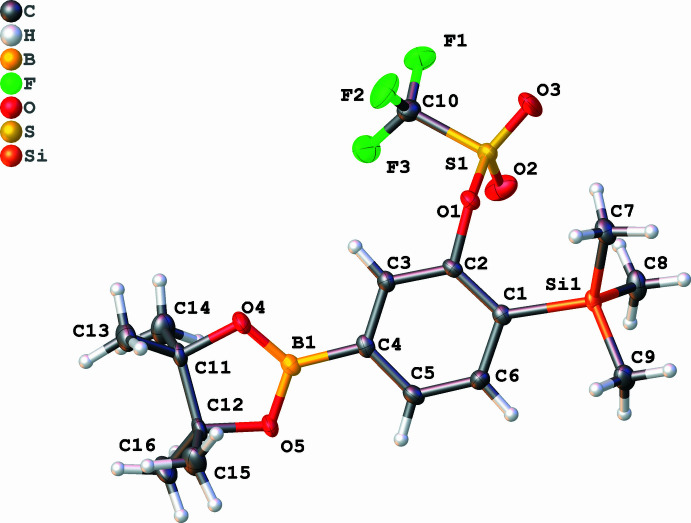
The mol­ecular structure of compound **1a**. Displacement ellipsoids are drawn at the 50% probability level.

**Figure 4:**
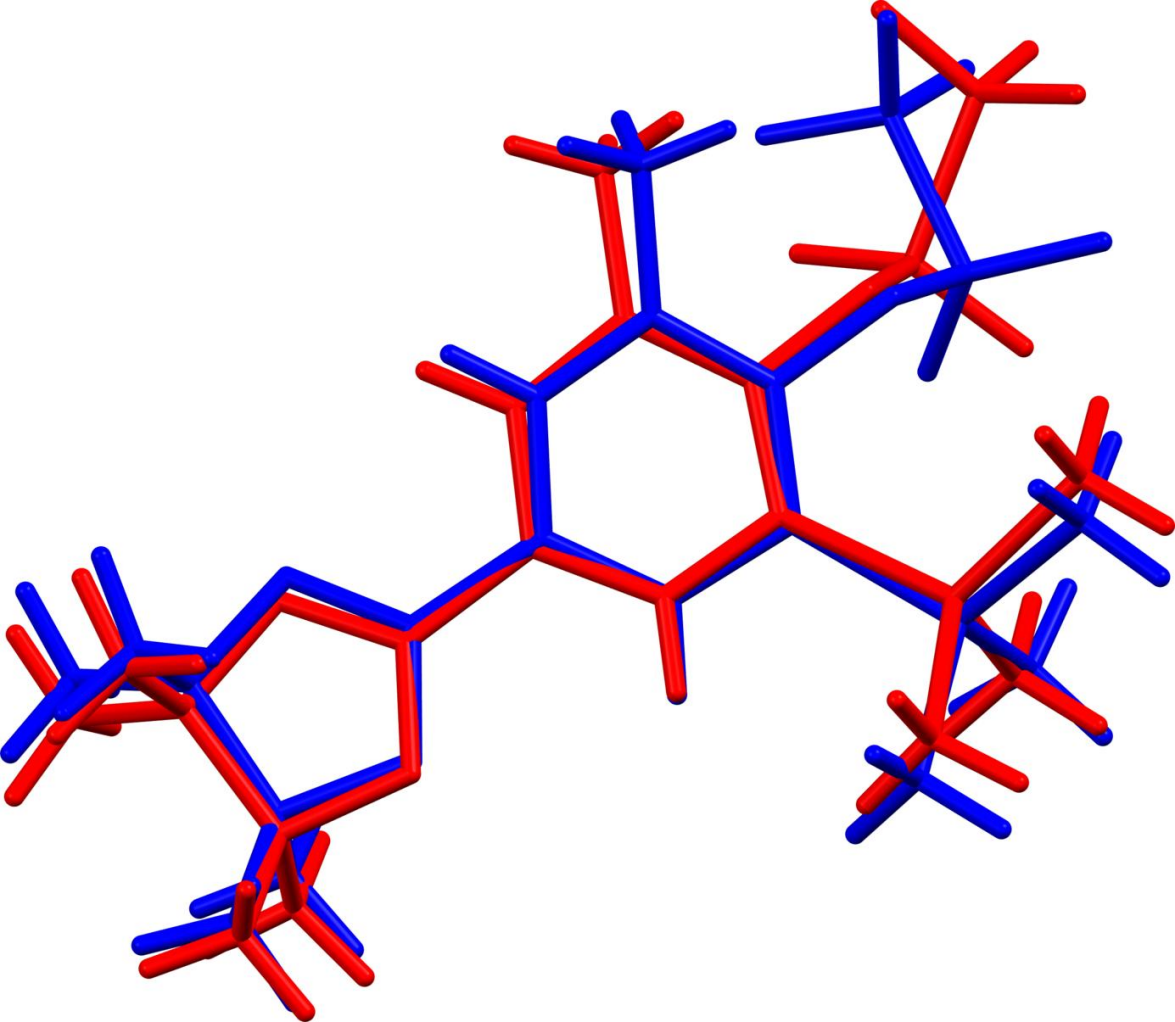
Overlay of the two independent mol­ecules of compound **2**.

Compound **1a** crystallizes in the ortho­rhom­bic space group *Pna*2_1_ with one mol­ecule in the asymmetric unit (*Z* = 4). The central ring and the directly attached heteratoms form a nearly planar motif (average deviation from the least squares plane = 0.062 Å). The C—B, C—Si, and C—O bond distances are within the expected values for single bonds: 1.572 (4), 1.909 (3) and 1.450 (3) Å, respectively. Compound **1b** crystallizes in the monoclinic space group *C*2*/c* as colourless blocks with one mol­ecule in the asymmetric unit. The central benzene ring and its direct heteroatom (O, Si, and B) form a nearly perfect plane (mean deviation from the least squares plane = 0.021 Å). The pinacolate moiety is disordered over two sites with site occupancy factors of 0.905 and 0.095, attached to one pivot borane atom. The C—B, C—Si, and C—O bonds are 1.599 (3), 1.908 (2), and 1.443 (2) Å, respectively, in the typical range for C*sp* ^2^—*E* single bonds. Compound **2** crystallizes in the monoclinic space group *P*2_1_ */n* (*Z* = 8) with two mol­ecules in the asymmetric unit of very similar metric parameters, except for the orientation of the triflate group [C2—O1—S1—C10 = 95.7 (3) and 150.1 (3)°], as shown in Fig. 4. The C—B, C—Si, and C—O bonds are 1.558 (5)/1.553 (5), 1.451 (4)/1.450 (4), and 1.908 (4)/1.899 (4) Å, respectively. It is noteworthy that the variation of the C—B bond length is the largest in this series, albeit still within the expected bond length for a carbon–boron single bond and within the respective standard deviations (see Table 1 ▸ and Figs. 1 ▸–4 ▸ ▸ ▸).

## 3. Supra­molecular features

**Figure 5:**
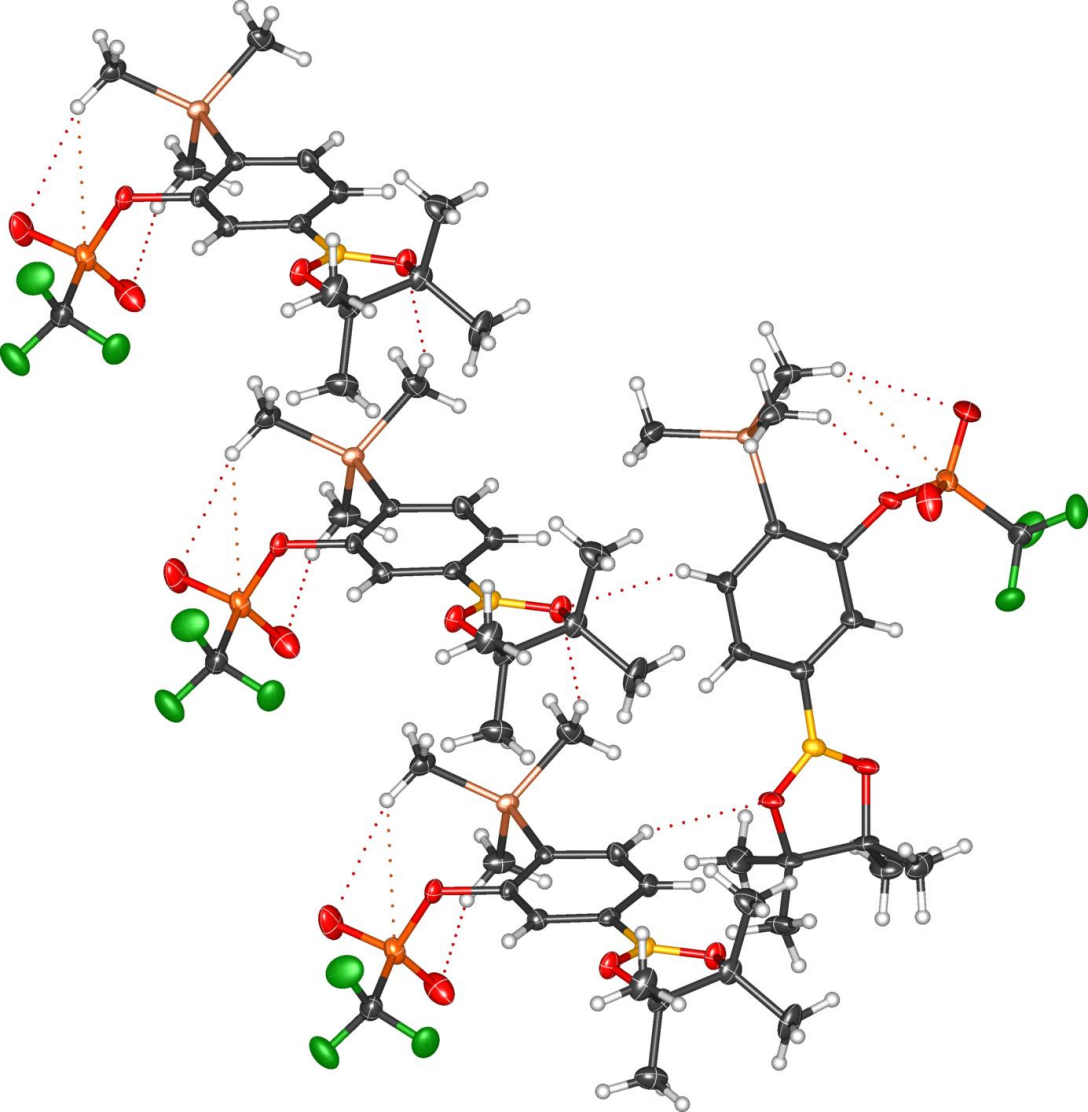
Solid-state packing of compound **1a** showing short inter- and intra­molecular inter­actions.

The supra­molecular arrangement of **1a**, **1b**, and **2** is unexceptional and shows only very weak inter­molecular ar­yl/methyl-H⋯O (>2.58 Å) and ar­yl/methyl-H⋯F (>2.60 Å) inter­actions, the latter being slightly below the sum of their van der Waals radii. In compound **1a**, the mol­ecular motifs arrange in a slipped manner giving a stair-like arrangement. Besides these weak Si(CH_3_)_3_⋯O inter­actions [2.797 (2) Å], further aryl-H⋯O inter­actions [2.683 (2) Å] dominate the packing. The crystal structure of **1b** is characterized by inter­molecular CH_3_⋯O inter­actions of two neighbouring pinacolborane units [H⋯O: 2.637 (2) Å] and weak F⋯π inter­actions [O⋯centroid: 3.574 (3) Å]. The major packing motif of **2** involves a head-to-tail arrangement of two symmetry-related mol­ecules resulting in weak CH_3_(pinacol)⋯O(triflate) inter­actions [2.632 (3) Å]. Supra­molecular features are illustrated in Figs. 5 ▸–7 ▸ ▸.

## 4. Database survey

A database survey (Cambridge Structural Database, WEBCSD v.1.9.40; Groom *et al.*, 2016 ▸) shows that, despite the large inter­est in these aryne precursors, only a limited number of *ortho*-silylaryl triflates have been structurally characterized, including precursors for complex natural products (Guo *et al.*, 2023 ▸: BEVBIR), polycyclic hydro­carbons (Dauvergne *et al.*, 2022 ▸: DIBPIR; Wu *et al.*, 2022 ▸: PAVBOH, PAVCIC; Elbert *et al.*, 2020 ▸: UVANUD; Tozawa *et al.*, 2017 ▸: WEDRUV), polymers (Xin *et al.*, 2019 ▸: LONCID) and others (Mochida *et al.*, 2009 ▸: UQAXIV; Haas *et al.*, 2022 ▸: XATROD). To the best of our knowledge, no borylated *ortho*-silylaryl triflates have been structurally characterized so far.

## 5. Synthesis and crystallization

Aryl boronates **1**–**2** were synthesised *via* Ir-catalysed C—H borylation according to a previously reported protocol (Demory *et al.*, 2015 ▸). Crystals of **1**–**2** were grown according to the following procedures:

**5-(4,4,5,5-Tetra­methyl-1,3,2-dioxaborolan-2-yl)-2-(tri­meth­yl­sil­yl)phenyl tri­fluoro­methane­sulfonate** (**1a**):

In a 100 mL round-bottom flask placed in an oil bath, **1a** (10.00 g, 23.56 mmol) was heated to 313 K and dissolved in a minimal amount of *n*-pentane. The solution was cooled to RT and then placed in a freezer (255 K) for 2 h. Crystals of **1a** formed as colourless shards, which were filtered and washed with ice-cold pentane.

**4-(4,4,5,5-Tetra­methyl-1,3,2-dioxaborolan-2-yl)-2-(tri­meth­yl­sil­yl)phenyl tri­fluoro­methane­sulfonate** (**1b**):

A 100 mL round-bottom flask containing a suspension of **1a** and **1b** (4.00 g, 9.42 mmol, **1a**/**1b** ≃ 2.5:1) in *n*-pentane (15 mL) was heated gently to 313 K and filtered through a sintered frit. The filtrate was concentrated under reduced pressure, giving a colourless solid. This procedure was repeated twice, yielding a viscous colourless oil, storage of which under air for six weeks at RT afforded cubic crystals of **1b**.

**2-Methyl-4-(4,4,5,5-tetra­methyl-1,3,2-dioxaborolan-2-yl)-6-(tri­methyl­sil­yl)phenyl tri­fluoro­methane­sulfonate** (**2**):

In a 25 mL round-bottom flask placed in an oil bath, **2** (0.20 g, 0.46 mmol) was heated to 313 K and dissolved in a minimal amount of *n*-pentane. The flask was stoppered and cooled in a freezer to 155 K over the course of 0.5 h. Cubic crystals of **2** (approx. 1 mm in width) formed as a suspension. These were separated from the mother liquor by filtration and then washed with cold (155 K) *n*-pentane.

## 6. Refinement

Crystal data, data collection and structure refinement details are summarized in Table 2 ▸. Compound **1b** was modelled with a positional disorder of the pinacolborane moiety pivoting around the boron atom. The site occupancy factors were freely refined to give a 0.097 (4):0.903 (4) occupancy.

Attempts to model the tri­fluoro­methane­sulfonate group in **1b** with a positional disorder using two (and three) parts, did not produced a satisfactory model. Hence the refinement with somewhat large ellipsoids for the tri­fluoro­methane­sulfonate group was finally used. Hydrogen atoms were refined with common isotropic displacement parameters for the H atoms of the same group and idealized geometries with methyl groups allowed to freely rotate about the C—C bond. The distances for methyl and aromatic C—H groups were set to 0.98 Å and 0.95 Å, respectively.

## Supplementary Material

Crystal structure: contains datablock(s) global, 1_a, 1b, 2. DOI: 10.1107/S2056989024000264/dj2074sup1.cif

Structure factors: contains datablock(s) 1_a. DOI: 10.1107/S2056989024000264/dj20741_asup6.hkl

Structure factors: contains datablock(s) 1b. DOI: 10.1107/S2056989024000264/dj20741bsup7.hkl

Structure factors: contains datablock(s) 2. DOI: 10.1107/S2056989024000264/dj20742sup8.hkl

Supporting information file. DOI: 10.1107/S2056989024000264/dj20741_asup5.cml

Supporting information file. DOI: 10.1107/S2056989024000264/dj20741bsup6.cml

Supporting information file. DOI: 10.1107/S2056989024000264/dj20742sup7.cml

CCDC references: 2324175, 2324174, 2324173

Additional supporting information:  crystallographic information; 3D view; checkCIF report

## Figures and Tables

**Figure 2:**
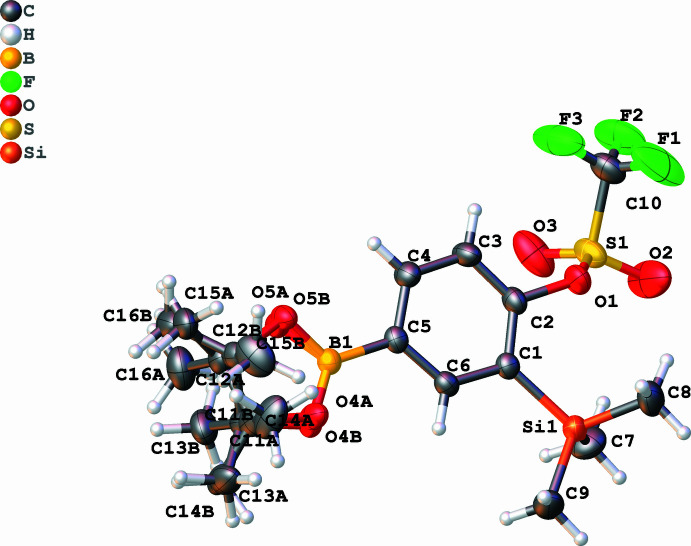
The mol­ecular structure of compound **1b**. Displacement ellipsoids are drawn at the 50% probability level.

**Figure 3:**
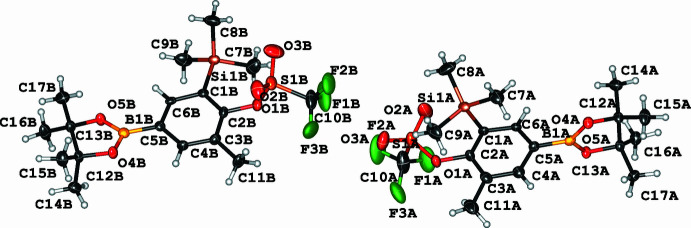
The two independent mol­ecules in compound **2**. Displacement ellipsoids are drawn at the 50% probability level.

**Figure 6:**
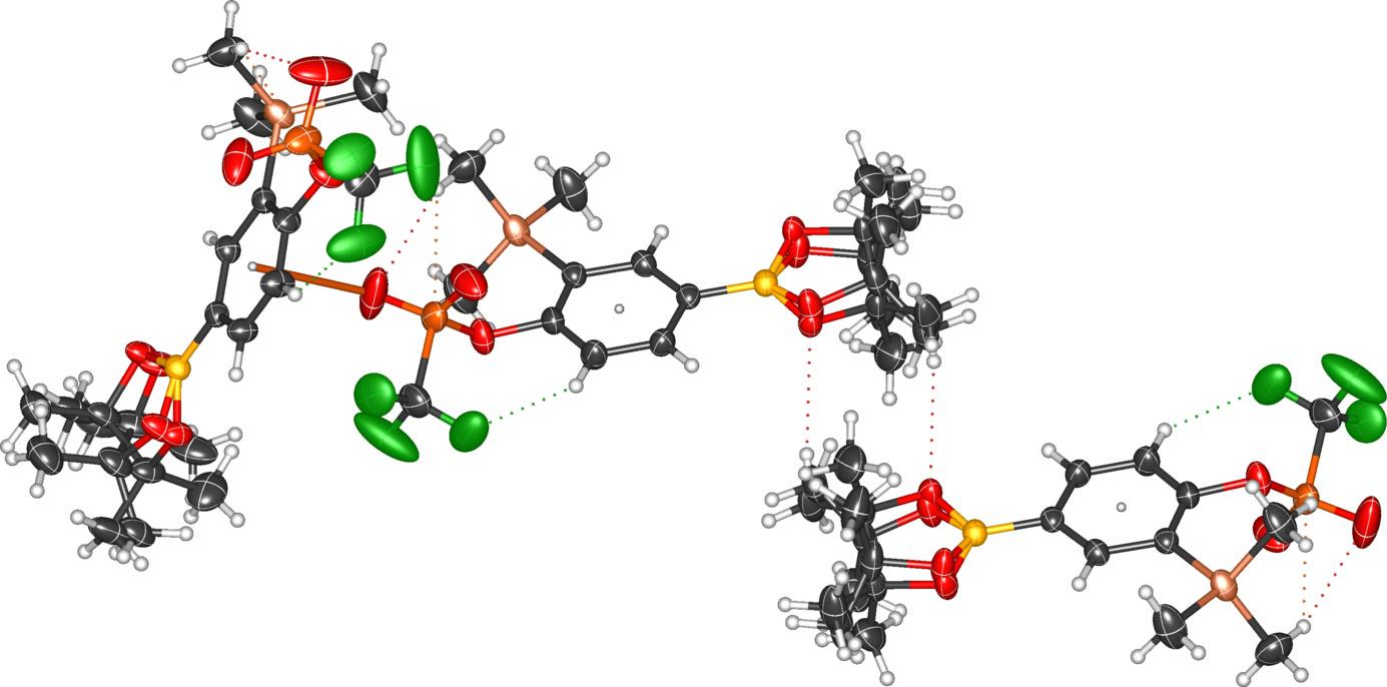
Solid-state packing of compound **1b** showing short inter- and intra­molecular inter­actions.

**Figure 7:**
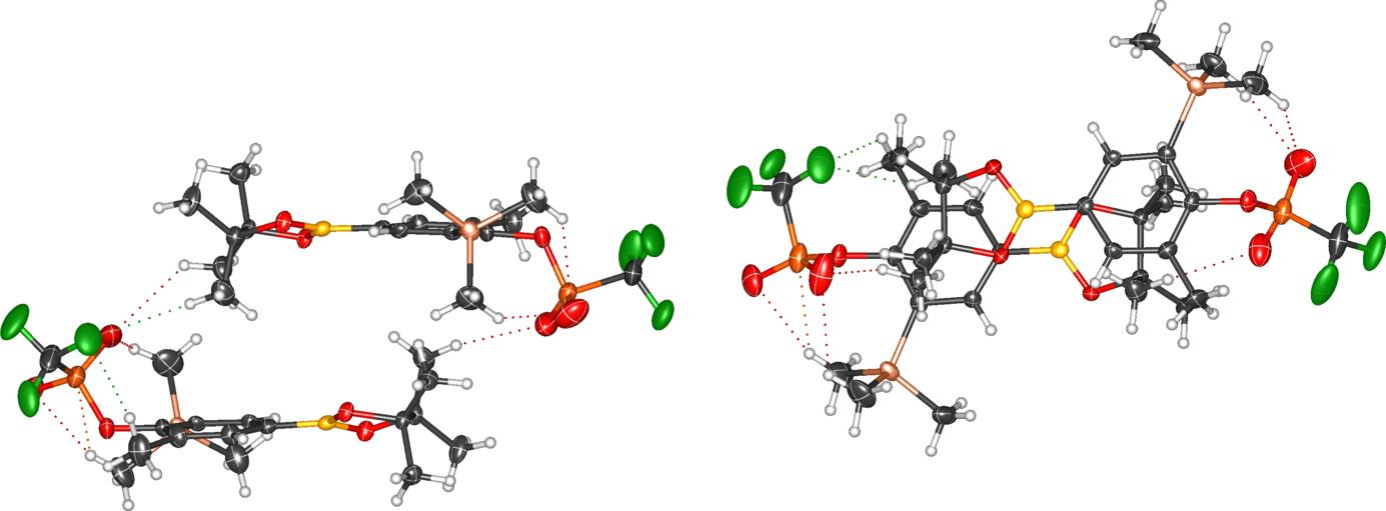
Solid-state packing of compound **2** showing short inter- and intra­molecular inter­actions.

**Table 1:** Selected bond distances (Å)

	**1a**	**1b**	**2**
C—B	1.572 (4)	1.599 (3)	1.558 (5) /1.553 (5)
C—O	1.450 (3)	1.443 (2)	1.451 (4) / 1.450 (4)
C—Si	1.909 (3)	1.908 (2)	1.908 (4) / 1.899 (4)

**Table 2:** Experimental details

	**1a**	**1b**	**2**
Crystal data			
Chemical formula	C_16_H_24_BF_3_O_5_SSi	C_16_H_24_BF_3_O_5_SSi	C_17_H_26_BF_3_O_5_SSi
*M*_r_	424.31	424.31	438.34
Crystal system, space group	Orthorhombic, *P* *n* *a*2_1_	Monoclinic, *C*2/*c*	Monoclinic, *P*2_1_/*n*
Temperature (K)	150	170	150
*a*, *b*, *c* (Å)	10.4316 (7), 25.1732 (17), 7.7756 (5)	28.334 (3), 11.8449 (13), 12.9139 (14)	10.2407 (8), 12.6295 (10), 34.127 (3)
α, β, γ (°)	90, 90, 90	90, 92.723 (2), 90	90, 95.689 (2), 90
*V* (Å^3^)	2041.8 (2)	4329.1 (8)	4392.1 (6)
*Z*	4	8	8
Radiation type	Mo *K*α	Mo *K*α	Mo *K*α
μ (mm^−1^)	0.27	0.25	0.25
Crystal size (mm)	0.3 × 0.22 × 0.18	0.13 × 0.13 × 0.12	0.32 × 0.15 × 0.1

Data collection			
Diffractometer	Bruker APEXII CCD	Bruker *APEX*-II CCD	Bruker *APEX*-II CCD
Absorption correction	Multi-scan (*SADABS*; Krause *et al.*, 2015 ▸)	Multi-scan (*SADABS*; Krause *et al.*, 2015 ▸)	Multi-scan (*SADABS*; Krause *et al.*, 2015 ▸)
*T*_min_, *T*_max_	0.680, 0.746	0.717, 0.746	0.639, 0.745
No. of measured, independent and observed [*I* > 2σ(*I*)] reflections	13726, 5205, 4654	44626, 5373, 4180	104215, 9044, 6597
*R*_int_	0.042	0.042	0.091

Refinement			
*R*[*F* ^2^ > 2σ(*F* ^2^)], *wR*(*F* ^2^), *S*	0.040, 0.091, 1.04	0.048, 0.139, 1.05	0.072, 0.186, 1.14
No. of reflections	5205	5373	9044
No. of parameters	251	328	521
No. of restraints	1	171	0
H-atom treatment	H-atom parameters constrained	H-atom parameters constrained	H-atom parameters constrained
Δρ_max_, Δρ_min_ (e Å^−3^)	0.31, −0.25	0.56, −0.36	0.60, −0.48
Absolute structure	Flack *x* determined using 1809 quotients [(*I* ^+^)−(*I* ^−^)]/[(*I* ^+^)+(*I* ^−^)] (Parsons *et al.*, 2013 ▸)	–	–
Absolute structure parameter	0.11 (5)	–	–

Computer programs: *APEX2* and *SAINT* (Bruker, 2013 ▸), *SHELXT2014/4* (Sheldrick, 2015*a* ▸), *SHELXL2018/3* (Sheldrick, 2015*b* ▸) and *OLEX2* (Dolomanov *et al.*, 2009 ▸).

## supplementary crystallographic information

### 5-(4,4,5,5-Tetramethyl-1,3,2-dioxaborolan-2-yl)-2-(trimethylsilyl)phenyl trifluoromethanesulfonate (1_a) Crystal data

**Table:**

C_16_H_24_BF_3_O_5_SSi	*D*_x_ = 1.380 Mg m^−^^3^
*M**_r_* = 424.31	Mo *K*α radiation, λ = 0.71073 Å
Orthorhombic, *P**n**a*2_1_	Cell parameters from 3068 reflections
*a* = 10.4316 (7) Å	θ = 2.5–26.6°
*b* = 25.1732 (17) Å	µ = 0.27 mm^−^^1^
*c* = 7.7756 (5) Å	*T* = 150 K
*V* = 2041.8 (2) Å^3^	Block, clear colourless
*Z* = 4	0.3 × 0.22 × 0.18 mm
*F*(000) = 888	

### 5-(4,4,5,5-Tetramethyl-1,3,2-dioxaborolan-2-yl)-2-(trimethylsilyl)phenyl trifluoromethanesulfonate (1_a) Data collection

**Table:**

Bruker APEXII CCD diffractometer	4654 reflections with *I* > 2σ(*I*)
φ and ω scans	*R*_int_ = 0.042
Absorption correction: multi-scan (SADABS; Krause *et al.*, 2015)	θ_max_ = 28.8°, θ_min_ = 1.6°
*T*_min_ = 0.680, *T*_max_ = 0.746	*h* = −13→14
13726 measured reflections	*k* = −34→23
5205 independent reflections	*l* = −9→10

### 5-(4,4,5,5-Tetramethyl-1,3,2-dioxaborolan-2-yl)-2-(trimethylsilyl)phenyl trifluoromethanesulfonate (1_a) Refinement

**Table:**

Refinement on *F*^2^	Secondary atom site location: difference Fourier map
Least-squares matrix: full	Hydrogen site location: inferred from neighbouring sites
*R*[*F*^2^ > 2σ(*F*^2^)] = 0.040	H-atom parameters constrained
*wR*(*F*^2^) = 0.091	*w* = 1/[σ^2^(*F*_o_^2^) + (0.0416*P*)^2^ + 0.1163*P*] where *P* = (*F*_o_^2^ + 2*F*_c_^2^)/3
*S* = 1.04	(Δ/σ)_max_ < 0.001
5205 reflections	Δρ_max_ = 0.31 e Å^−^^3^
251 parameters	Δρ_min_ = −0.25 e Å^−^^3^
1 restraint	Absolute structure: Flack *x* determined using 1809 quotients [(*I*^+^)-(*I*^-^)]/[(*I*^+^)+(*I*^-^)] (Parsons *et al.*, 2013)
Primary atom site location: structure-invariant direct methods	Absolute structure parameter: 0.11 (5)

### 5-(4,4,5,5-Tetramethyl-1,3,2-dioxaborolan-2-yl)-2-(trimethylsilyl)phenyl trifluoromethanesulfonate (1_a) Special details

**Table:**

Geometry. All esds (except the esd in the dihedral angle between two l.s. planes) are estimated using the full covariance matrix. The cell esds are taken into account individually in the estimation of esds in distances, angles and torsion angles; correlations between esds in cell parameters are only used when they are defined by crystal symmetry. An approximate (isotropic) treatment of cell esds is used for estimating esds involving l.s. planes.

### 5-(4,4,5,5-Tetramethyl-1,3,2-dioxaborolan-2-yl)-2-(trimethylsilyl)phenyl trifluoromethanesulfonate (1_a) Fractional atomic coordinates and isotropic or equivalent isotropic displacement parameters (Å^2^)

**Table:**

	*x*	*y*	*z*	*U*_iso_*/*U*_eq_	
S1	1.01340 (7)	0.66139 (3)	0.65946 (11)	0.02312 (17)	
Si1	0.84369 (7)	0.55136 (3)	0.93641 (11)	0.01775 (17)	
O4	0.5211 (2)	0.67505 (8)	0.2262 (3)	0.0228 (5)	
O5	0.45562 (19)	0.58908 (8)	0.1909 (3)	0.0222 (4)	
O1	0.86972 (19)	0.66011 (7)	0.7168 (3)	0.0196 (4)	
F3	0.9583 (2)	0.69871 (8)	0.3559 (3)	0.0431 (6)	
F2	0.9483 (2)	0.75640 (8)	0.5581 (3)	0.0450 (6)	
F1	1.13163 (19)	0.72872 (8)	0.4680 (3)	0.0430 (6)	
O2	1.0494 (2)	0.61447 (9)	0.5732 (4)	0.0373 (6)	
O3	1.0836 (2)	0.68223 (10)	0.8003 (3)	0.0390 (6)	
C2	0.7803 (2)	0.62207 (11)	0.6445 (4)	0.0170 (5)	
C1	0.7643 (3)	0.57397 (11)	0.7276 (4)	0.0165 (5)	
C11	0.4470 (3)	0.67413 (12)	0.0647 (4)	0.0212 (6)	
C6	0.6722 (3)	0.54073 (11)	0.6516 (4)	0.0211 (6)	
H6	0.657274	0.506655	0.700137	0.025*	
C12	0.3703 (3)	0.62130 (12)	0.0824 (4)	0.0217 (6)	
C9	0.7285 (3)	0.50567 (13)	1.0438 (4)	0.0281 (7)	
H9A	0.720683	0.472937	0.976334	0.042*	
H9B	0.644498	0.522905	1.052125	0.042*	
H9C	0.759667	0.497095	1.159385	0.042*	
C5	0.6021 (3)	0.55591 (12)	0.5078 (4)	0.0208 (6)	
H5	0.542556	0.531702	0.458700	0.025*	
C3	0.7097 (3)	0.63938 (11)	0.5050 (4)	0.0179 (6)	
H3	0.724025	0.673676	0.457729	0.021*	
C4	0.6174 (2)	0.60599 (11)	0.4345 (4)	0.0179 (5)	
C7	0.8752 (3)	0.60863 (12)	1.0824 (4)	0.0253 (7)	
H7A	0.798144	0.630777	1.090236	0.038*	
H7B	0.946258	0.629812	1.036707	0.038*	
H7C	0.897695	0.595400	1.197029	0.038*	
C10	1.0115 (3)	0.71493 (13)	0.4994 (4)	0.0261 (7)	
C13	0.3650 (3)	0.72370 (12)	0.0530 (5)	0.0317 (8)	
H13A	0.311296	0.726531	0.155987	0.048*	
H13B	0.310190	0.721590	−0.049200	0.048*	
H13C	0.420394	0.755027	0.044514	0.048*	
C8	0.9948 (3)	0.51573 (13)	0.8842 (4)	0.0281 (7)	
H8A	1.034537	0.502920	0.990621	0.042*	
H8B	1.053749	0.539992	0.825421	0.042*	
H8C	0.975750	0.485481	0.809093	0.042*	
C14	0.5449 (3)	0.67315 (15)	−0.0799 (5)	0.0329 (8)	
H14A	0.600916	0.704253	−0.070567	0.049*	
H14B	0.500365	0.673849	−0.190873	0.049*	
H14C	0.596418	0.640708	−0.071530	0.049*	
C15	0.2458 (3)	0.62724 (15)	0.1819 (5)	0.0352 (8)	
H15A	0.210566	0.591997	0.207024	0.053*	
H15B	0.184177	0.647459	0.112863	0.053*	
H15C	0.262362	0.646087	0.289887	0.053*	
C16	0.3481 (4)	0.59221 (13)	−0.0848 (5)	0.0360 (9)	
H16A	0.430777	0.583498	−0.137516	0.054*	
H16B	0.298674	0.614841	−0.163079	0.054*	
H16C	0.300326	0.559400	−0.062388	0.054*	
B1	0.5305 (3)	0.62397 (14)	0.2799 (4)	0.0190 (7)	

### 5-(4,4,5,5-Tetramethyl-1,3,2-dioxaborolan-2-yl)-2-(trimethylsilyl)phenyl trifluoromethanesulfonate (1_a) Atomic displacement parameters (Å^2^)

**Table:**

	*U*^11^	*U*^22^	*U*^33^	*U*^12^	*U*^13^	*U*^23^
S1	0.0194 (3)	0.0228 (3)	0.0272 (4)	−0.0035 (3)	−0.0057 (3)	0.0051 (3)
Si1	0.0167 (3)	0.0241 (4)	0.0125 (3)	0.0017 (3)	−0.0021 (3)	0.0018 (3)
O4	0.0244 (11)	0.0242 (11)	0.0198 (10)	−0.0003 (9)	−0.0106 (9)	−0.0009 (9)
O5	0.0236 (10)	0.0230 (10)	0.0202 (11)	0.0003 (8)	−0.0110 (9)	0.0018 (9)
O1	0.0202 (9)	0.0206 (10)	0.0180 (10)	−0.0023 (8)	−0.0062 (8)	−0.0016 (8)
F3	0.0592 (14)	0.0485 (13)	0.0215 (10)	−0.0185 (11)	−0.0091 (10)	0.0045 (10)
F2	0.0694 (15)	0.0230 (10)	0.0426 (13)	0.0106 (10)	0.0058 (12)	0.0066 (10)
F1	0.0371 (11)	0.0424 (12)	0.0495 (15)	−0.0163 (9)	0.0026 (10)	0.0122 (10)
O2	0.0287 (12)	0.0236 (12)	0.0595 (17)	0.0027 (9)	0.0093 (12)	0.0017 (12)
O3	0.0298 (12)	0.0516 (16)	0.0356 (14)	−0.0156 (12)	−0.0179 (11)	0.0106 (12)
C2	0.0138 (12)	0.0199 (13)	0.0172 (13)	−0.0007 (10)	−0.0020 (12)	−0.0033 (12)
C1	0.0158 (12)	0.0190 (13)	0.0145 (13)	0.0017 (11)	0.0006 (11)	−0.0019 (11)
C11	0.0238 (15)	0.0256 (15)	0.0144 (14)	0.0033 (12)	−0.0080 (13)	0.0015 (12)
C6	0.0214 (13)	0.0204 (13)	0.0216 (14)	−0.0032 (11)	−0.0024 (13)	0.0041 (13)
C12	0.0199 (14)	0.0259 (15)	0.0193 (14)	0.0040 (12)	−0.0100 (12)	0.0014 (12)
C9	0.0270 (16)	0.0381 (19)	0.0194 (15)	−0.0042 (13)	−0.0019 (13)	0.0073 (14)
C5	0.0182 (13)	0.0249 (15)	0.0193 (14)	−0.0033 (11)	−0.0035 (12)	−0.0019 (12)
C3	0.0166 (13)	0.0200 (14)	0.0170 (13)	0.0022 (11)	−0.0022 (11)	0.0002 (12)
C4	0.0152 (11)	0.0241 (14)	0.0144 (12)	0.0039 (10)	0.0002 (12)	−0.0013 (12)
C7	0.0273 (15)	0.0327 (17)	0.0161 (14)	0.0025 (13)	−0.0035 (12)	−0.0025 (13)
C10	0.0313 (16)	0.0244 (16)	0.0227 (16)	−0.0047 (13)	−0.0021 (13)	−0.0006 (13)
C13	0.0355 (17)	0.0281 (17)	0.0315 (18)	0.0094 (14)	−0.0131 (16)	−0.0017 (15)
C8	0.0239 (15)	0.0389 (19)	0.0215 (16)	0.0099 (14)	0.0008 (12)	0.0044 (14)
C14	0.0310 (17)	0.044 (2)	0.0239 (17)	0.0036 (14)	0.0043 (15)	0.0072 (16)
C15	0.0187 (14)	0.049 (2)	0.038 (2)	0.0012 (14)	−0.0016 (15)	0.0097 (17)
C16	0.047 (2)	0.0318 (18)	0.029 (2)	0.0002 (15)	−0.0207 (17)	−0.0064 (15)
B1	0.0168 (14)	0.0240 (17)	0.0161 (15)	0.0010 (13)	−0.0014 (13)	−0.0028 (13)

### 5-(4,4,5,5-Tetramethyl-1,3,2-dioxaborolan-2-yl)-2-(trimethylsilyl)phenyl trifluoromethanesulfonate (1_a) Geometric parameters (Å, º)

**Table:**

S1—O1	1.564 (2)	C9—H9A	0.9800
S1—O2	1.409 (3)	C9—H9B	0.9800
S1—O3	1.418 (2)	C9—H9C	0.9800
S1—C10	1.834 (3)	C5—H5	0.9500
Si1—C1	1.909 (3)	C5—C4	1.393 (4)
Si1—C9	1.861 (3)	C3—H3	0.9500
Si1—C7	1.864 (3)	C3—C4	1.390 (4)
Si1—C8	1.858 (3)	C4—B1	1.572 (4)
O4—C11	1.474 (3)	C7—H7A	0.9800
O4—B1	1.356 (4)	C7—H7B	0.9800
O5—C12	1.470 (3)	C7—H7C	0.9800
O5—B1	1.364 (4)	C13—H13A	0.9800
O1—C2	1.450 (3)	C13—H13B	0.9800
F3—C10	1.311 (4)	C13—H13C	0.9800
F2—C10	1.316 (4)	C8—H8A	0.9800
F1—C10	1.323 (4)	C8—H8B	0.9800
C2—C1	1.383 (4)	C8—H8C	0.9800
C2—C3	1.382 (4)	C14—H14A	0.9800
C1—C6	1.405 (4)	C14—H14B	0.9800
C11—C12	1.558 (4)	C14—H14C	0.9800
C11—C13	1.516 (4)	C15—H15A	0.9800
C11—C14	1.518 (5)	C15—H15B	0.9800
C6—H6	0.9500	C15—H15C	0.9800
C6—C5	1.389 (4)	C16—H16A	0.9800
C12—C15	1.519 (4)	C16—H16B	0.9800
C12—C16	1.511 (4)	C16—H16C	0.9800
			
O1—S1—C10	101.43 (13)	C5—C4—B1	120.5 (3)
O2—S1—O1	111.95 (13)	C3—C4—C5	117.7 (3)
O2—S1—O3	122.67 (16)	C3—C4—B1	121.8 (3)
O2—S1—C10	107.20 (16)	Si1—C7—H7A	109.5
O3—S1—O1	106.41 (14)	Si1—C7—H7B	109.5
O3—S1—C10	104.94 (14)	Si1—C7—H7C	109.5
C9—Si1—C1	106.59 (13)	H7A—C7—H7B	109.5
C9—Si1—C7	108.58 (16)	H7A—C7—H7C	109.5
C7—Si1—C1	111.33 (13)	H7B—C7—H7C	109.5
C8—Si1—C1	109.03 (14)	F3—C10—S1	110.7 (2)
C8—Si1—C9	110.32 (15)	F3—C10—F2	109.3 (3)
C8—Si1—C7	110.91 (15)	F3—C10—F1	109.0 (3)
B1—O4—C11	106.6 (2)	F2—C10—S1	110.7 (2)
B1—O5—C12	106.4 (2)	F2—C10—F1	109.3 (3)
C2—O1—S1	121.32 (17)	F1—C10—S1	107.9 (2)
C1—C2—O1	118.3 (2)	C11—C13—H13A	109.5
C3—C2—O1	116.0 (2)	C11—C13—H13B	109.5
C3—C2—C1	125.4 (3)	C11—C13—H13C	109.5
C2—C1—Si1	127.3 (2)	H13A—C13—H13B	109.5
C2—C1—C6	114.0 (2)	H13A—C13—H13C	109.5
C6—C1—Si1	118.5 (2)	H13B—C13—H13C	109.5
O4—C11—C12	102.0 (2)	Si1—C8—H8A	109.5
O4—C11—C13	109.5 (2)	Si1—C8—H8B	109.5
O4—C11—C14	106.2 (2)	Si1—C8—H8C	109.5
C13—C11—C12	114.7 (2)	H8A—C8—H8B	109.5
C13—C11—C14	110.4 (3)	H8A—C8—H8C	109.5
C14—C11—C12	113.4 (3)	H8B—C8—H8C	109.5
C1—C6—H6	118.8	C11—C14—H14A	109.5
C5—C6—C1	122.3 (3)	C11—C14—H14B	109.5
C5—C6—H6	118.8	C11—C14—H14C	109.5
O5—C12—C11	102.2 (2)	H14A—C14—H14B	109.5
O5—C12—C15	106.3 (2)	H14A—C14—H14C	109.5
O5—C12—C16	108.6 (2)	H14B—C14—H14C	109.5
C15—C12—C11	113.6 (3)	C12—C15—H15A	109.5
C16—C12—C11	114.6 (3)	C12—C15—H15B	109.5
C16—C12—C15	110.8 (3)	C12—C15—H15C	109.5
Si1—C9—H9A	109.5	H15A—C15—H15B	109.5
Si1—C9—H9B	109.5	H15A—C15—H15C	109.5
Si1—C9—H9C	109.5	H15B—C15—H15C	109.5
H9A—C9—H9B	109.5	C12—C16—H16A	109.5
H9A—C9—H9C	109.5	C12—C16—H16B	109.5
H9B—C9—H9C	109.5	C12—C16—H16C	109.5
C6—C5—H5	119.4	H16A—C16—H16B	109.5
C6—C5—C4	121.2 (3)	H16A—C16—H16C	109.5
C4—C5—H5	119.4	H16B—C16—H16C	109.5
C2—C3—H3	120.4	O4—B1—O5	114.4 (3)
C2—C3—C4	119.2 (3)	O4—B1—C4	123.4 (3)
C4—C3—H3	120.4	O5—B1—C4	122.2 (3)
			
S1—O1—C2—C1	91.5 (3)	C11—O4—B1—O5	−9.8 (3)
S1—O1—C2—C3	−93.9 (3)	C11—O4—B1—C4	171.6 (3)
Si1—C1—C6—C5	174.5 (2)	C6—C5—C4—C3	3.0 (4)
O4—C11—C12—O5	−28.5 (3)	C6—C5—C4—B1	−175.7 (3)
O4—C11—C12—C15	85.5 (3)	C12—O5—B1—O4	−9.9 (3)
O4—C11—C12—C16	−145.7 (3)	C12—O5—B1—C4	168.7 (3)
O1—S1—C10—F3	−74.9 (2)	C5—C4—B1—O4	165.7 (3)
O1—S1—C10—F2	46.4 (2)	C5—C4—B1—O5	−12.8 (4)
O1—S1—C10—F1	165.9 (2)	C3—C2—C1—Si1	−171.8 (2)
O1—C2—C1—Si1	2.2 (4)	C3—C2—C1—C6	3.7 (4)
O1—C2—C1—C6	177.7 (2)	C3—C4—B1—O4	−13.0 (5)
O1—C2—C3—C4	−176.8 (2)	C3—C4—B1—O5	168.5 (3)
O2—S1—O1—C2	−16.3 (2)	C10—S1—O1—C2	97.7 (2)
O2—S1—C10—F3	42.6 (3)	C13—C11—C12—O5	−146.7 (3)
O2—S1—C10—F2	163.9 (2)	C13—C11—C12—C15	−32.7 (4)
O2—S1—C10—F1	−76.5 (2)	C13—C11—C12—C16	96.0 (3)
O3—S1—O1—C2	−152.8 (2)	C14—C11—C12—O5	85.2 (3)
O3—S1—C10—F3	174.5 (2)	C14—C11—C12—C15	−160.8 (3)
O3—S1—C10—F2	−64.2 (3)	C14—C11—C12—C16	−32.0 (4)
O3—S1—C10—F1	55.3 (3)	B1—O4—C11—C12	23.7 (3)
C2—C1—C6—C5	−1.4 (4)	B1—O4—C11—C13	145.5 (3)
C2—C3—C4—C5	−0.9 (4)	B1—O4—C11—C14	−95.3 (3)
C2—C3—C4—B1	177.8 (3)	B1—O5—C12—C11	23.8 (3)
C1—C2—C3—C4	−2.6 (4)	B1—O5—C12—C15	−95.5 (3)
C1—C6—C5—C4	−1.9 (5)	B1—O5—C12—C16	145.2 (3)

### 4-(4,4,5,5-Tetramethyl-1,3,2-dioxaborolan-2-yl)-2-(trimethylsilyl)phenyl trifluoromethanesulfonate (1b) Crystal data

**Table:**

C_16_H_24_BF_3_O_5_SSi	*F*(000) = 1776
*M**_r_* = 424.31	*D*_x_ = 1.302 Mg m^−^^3^
Monoclinic, *C*2/*c*	Mo *K*α radiation, λ = 0.71073 Å
*a* = 28.334 (3) Å	Cell parameters from 9969 reflections
*b* = 11.8449 (13) Å	θ = 2.4–28.3°
*c* = 12.9139 (14) Å	µ = 0.25 mm^−^^1^
β = 92.723 (2)°	*T* = 170 K
*V* = 4329.1 (8) Å^3^	Block, clear colourless
*Z* = 8	0.13 × 0.13 × 0.12 mm

### 4-(4,4,5,5-Tetramethyl-1,3,2-dioxaborolan-2-yl)-2-(trimethylsilyl)phenyl trifluoromethanesulfonate (1b) Data collection

**Table:**

Bruker APEX-II CCD diffractometer	4180 reflections with *I* > 2σ(*I*)
φ and ω scans	*R*_int_ = 0.042
Absorption correction: multi-scan (SADABS; Krause *et al.*, 2015)	θ_max_ = 28.3°, θ_min_ = 1.9°
*T*_min_ = 0.717, *T*_max_ = 0.746	*h* = −37→37
44626 measured reflections	*k* = −15→15
5373 independent reflections	*l* = −17→17

### 4-(4,4,5,5-Tetramethyl-1,3,2-dioxaborolan-2-yl)-2-(trimethylsilyl)phenyl trifluoromethanesulfonate (1b) Refinement

**Table:**

Refinement on *F*^2^	Primary atom site location: structure-invariant direct methods
Least-squares matrix: full	Secondary atom site location: difference Fourier map
*R*[*F*^2^ > 2σ(*F*^2^)] = 0.048	Hydrogen site location: inferred from neighbouring sites
*wR*(*F*^2^) = 0.139	H-atom parameters constrained
*S* = 1.05	*w* = 1/[σ^2^(*F*_o_^2^) + (0.0645*P*)^2^ + 4.7508*P*] where *P* = (*F*_o_^2^ + 2*F*_c_^2^)/3
5373 reflections	(Δ/σ)_max_ = 0.001
328 parameters	Δρ_max_ = 0.56 e Å^−^^3^
171 restraints	Δρ_min_ = −0.36 e Å^−^^3^

### 4-(4,4,5,5-Tetramethyl-1,3,2-dioxaborolan-2-yl)-2-(trimethylsilyl)phenyl trifluoromethanesulfonate (1b) Special details

**Table:**

Geometry. All esds (except the esd in the dihedral angle between two l.s. planes) are estimated using the full covariance matrix. The cell esds are taken into account individually in the estimation of esds in distances, angles and torsion angles; correlations between esds in cell parameters are only used when they are defined by crystal symmetry. An approximate (isotropic) treatment of cell esds is used for estimating esds involving l.s. planes.

### 4-(4,4,5,5-Tetramethyl-1,3,2-dioxaborolan-2-yl)-2-(trimethylsilyl)phenyl trifluoromethanesulfonate (1b) Fractional atomic coordinates and isotropic or equivalent isotropic displacement parameters (Å^2^)

**Table:**

	*x*	*y*	*z*	*U*_iso_*/*U*_eq_	Occ. (<1)
S1	0.39267 (2)	0.53526 (5)	0.90754 (5)	0.04790 (17)	
Si1	0.28275 (2)	0.42873 (4)	0.71167 (5)	0.03740 (15)	
O5B	0.43496 (8)	0.02880 (19)	0.5736 (2)	0.0351 (5)	0.903 (4)
O4B	0.35390 (7)	0.00957 (18)	0.57175 (19)	0.0369 (5)	0.903 (4)
O1	0.38245 (5)	0.51171 (12)	0.78959 (12)	0.0442 (4)	
F2	0.46272 (6)	0.64218 (17)	0.99534 (13)	0.0846 (6)	
F3	0.48082 (6)	0.55425 (18)	0.86149 (16)	0.0944 (7)	
O3	0.40303 (8)	0.43500 (16)	0.96328 (14)	0.0731 (6)	
C6	0.34638 (6)	0.24848 (15)	0.66026 (13)	0.0300 (4)	
H6	0.318434	0.212883	0.632970	0.036*	
C5	0.38952 (6)	0.19229 (14)	0.65209 (13)	0.0286 (4)	
C1	0.34246 (6)	0.35492 (14)	0.70687 (13)	0.0290 (4)	
C2	0.38459 (7)	0.39985 (15)	0.74578 (15)	0.0335 (4)	
C4	0.43043 (7)	0.24455 (16)	0.69264 (15)	0.0356 (4)	
H4	0.460103	0.208158	0.687406	0.043*	
O2	0.35905 (7)	0.6142 (2)	0.9391 (2)	0.0953 (8)	
F1	0.44191 (9)	0.70485 (18)	0.8471 (2)	0.1268 (10)	
C3	0.42828 (7)	0.34885 (17)	0.74042 (17)	0.0400 (5)	
H3	0.456046	0.384398	0.768729	0.048*	
B1	0.39244 (7)	0.07486 (17)	0.59818 (16)	0.0295 (4)	
C8	0.28793 (10)	0.57681 (19)	0.6644 (2)	0.0584 (6)	
H8A	0.308448	0.619943	0.713117	0.088*	
H8B	0.256540	0.611666	0.659150	0.088*	
H8C	0.301529	0.576629	0.595996	0.088*	
C7	0.26378 (9)	0.4261 (2)	0.8470 (2)	0.0590 (7)	
H7A	0.263471	0.347924	0.871953	0.089*	
H7B	0.231984	0.458201	0.849586	0.089*	
H7C	0.285849	0.470632	0.891142	0.089*	
C11B	0.37310 (8)	−0.10017 (19)	0.5400 (2)	0.0386 (5)	0.903 (4)
C9	0.23972 (9)	0.3503 (2)	0.6250 (2)	0.0633 (7)	
H9A	0.249285	0.355572	0.553247	0.095*	
H9B	0.208180	0.383126	0.630222	0.095*	
H9C	0.239064	0.270786	0.646029	0.095*	
C12B	0.42315 (8)	−0.0675 (2)	0.5057 (2)	0.0403 (5)	0.903 (4)
C10	0.44796 (9)	0.6143 (2)	0.90158 (19)	0.0526 (6)	
C16B	0.46034 (11)	−0.1584 (3)	0.5218 (3)	0.0599 (9)	0.903 (4)
H16A	0.490330	−0.131844	0.495879	0.090*	0.903 (4)
H16B	0.450216	−0.226705	0.484072	0.090*	0.903 (4)
H16C	0.464533	−0.175596	0.595905	0.090*	0.903 (4)
C14B	0.34042 (12)	−0.1493 (3)	0.4542 (3)	0.0602 (9)	0.903 (4)
H14A	0.309443	−0.165157	0.481598	0.090*	0.903 (4)
H14B	0.354036	−0.219480	0.428596	0.090*	0.903 (4)
H14C	0.336783	−0.094939	0.397162	0.090*	0.903 (4)
C13B	0.37450 (10)	−0.1748 (2)	0.6353 (2)	0.0558 (7)	0.903 (4)
H13A	0.396785	−0.143255	0.688037	0.084*	0.903 (4)
H13B	0.384702	−0.250861	0.616674	0.084*	0.903 (4)
H13C	0.342922	−0.178500	0.662994	0.084*	0.903 (4)
C15B	0.42273 (13)	−0.0214 (3)	0.3952 (2)	0.0667 (9)	0.903 (4)
H15A	0.398674	0.037744	0.386794	0.100*	0.903 (4)
H15B	0.415434	−0.082664	0.345989	0.100*	0.903 (4)
H15C	0.453822	0.010239	0.381897	0.100*	0.903 (4)
O4A	0.3600 (7)	0.023 (2)	0.547 (2)	0.044 (4)	0.097 (4)
O5A	0.4304 (8)	0.0112 (18)	0.594 (2)	0.046 (5)	0.097 (4)
C12A	0.4213 (7)	−0.0996 (16)	0.5538 (18)	0.045 (3)	0.097 (4)
C11A	0.3740 (7)	−0.0733 (18)	0.4901 (17)	0.051 (4)	0.097 (4)
C14A	0.3772 (10)	−0.030 (2)	0.3808 (15)	0.067 (6)	0.097 (4)
H14D	0.346748	−0.040496	0.342803	0.101*	0.097 (4)
H14E	0.401827	−0.070936	0.346002	0.101*	0.097 (4)
H14F	0.384976	0.051001	0.382771	0.101*	0.097 (4)
C16A	0.4204 (10)	−0.179 (2)	0.6455 (18)	0.066 (6)	0.097 (4)
H16D	0.452617	−0.203722	0.664381	0.099*	0.097 (4)
H16E	0.400645	−0.244226	0.627282	0.099*	0.097 (4)
H16F	0.407353	−0.139095	0.704417	0.099*	0.097 (4)
C15A	0.4579 (9)	−0.128 (2)	0.483 (2)	0.051 (5)	0.097 (4)
H15D	0.463707	−0.062548	0.438170	0.077*	0.097 (4)
H15E	0.447647	−0.192048	0.439586	0.077*	0.097 (4)
H15F	0.487125	−0.147289	0.522353	0.077*	0.097 (4)
C13A	0.3402 (9)	−0.164 (2)	0.502 (3)	0.060 (6)	0.097 (4)
H13D	0.338548	−0.182700	0.575078	0.090*	0.097 (4)
H13E	0.350261	−0.229980	0.463084	0.090*	0.097 (4)
H13F	0.309055	−0.138989	0.474168	0.090*	0.097 (4)

### 4-(4,4,5,5-Tetramethyl-1,3,2-dioxaborolan-2-yl)-2-(trimethylsilyl)phenyl trifluoromethanesulfonate (1b) Atomic displacement parameters (Å^2^)

**Table:**

	*U*^11^	*U*^22^	*U*^33^	*U*^12^	*U*^13^	*U*^23^
S1	0.0474 (3)	0.0458 (3)	0.0514 (3)	−0.0123 (2)	0.0120 (2)	−0.0229 (2)
Si1	0.0328 (3)	0.0282 (3)	0.0505 (3)	0.0018 (2)	−0.0045 (2)	−0.0038 (2)
O5B	0.0295 (7)	0.0330 (9)	0.0433 (10)	−0.0030 (6)	0.0051 (7)	−0.0123 (8)
O4B	0.0282 (8)	0.0277 (8)	0.0551 (13)	0.0027 (6)	0.0039 (7)	−0.0112 (8)
O1	0.0452 (8)	0.0332 (7)	0.0536 (9)	−0.0006 (6)	−0.0042 (7)	−0.0165 (6)
F2	0.0823 (12)	0.1048 (14)	0.0658 (10)	−0.0355 (10)	−0.0041 (8)	−0.0400 (10)
F3	0.0573 (10)	0.1180 (16)	0.1100 (15)	−0.0340 (10)	0.0253 (10)	−0.0636 (13)
O3	0.1234 (18)	0.0539 (11)	0.0433 (9)	−0.0297 (11)	0.0178 (10)	−0.0027 (8)
C6	0.0333 (9)	0.0267 (8)	0.0297 (9)	−0.0028 (7)	−0.0007 (7)	−0.0011 (7)
C5	0.0355 (9)	0.0260 (8)	0.0246 (8)	−0.0004 (7)	0.0039 (7)	−0.0003 (6)
C1	0.0339 (9)	0.0261 (8)	0.0270 (8)	−0.0002 (7)	0.0025 (7)	0.0004 (7)
C2	0.0383 (9)	0.0270 (8)	0.0356 (9)	−0.0032 (7)	0.0063 (8)	−0.0071 (7)
C4	0.0308 (9)	0.0355 (10)	0.0411 (10)	0.0000 (7)	0.0067 (7)	−0.0072 (8)
O2	0.0619 (12)	0.1032 (17)	0.1217 (19)	0.0035 (11)	0.0138 (12)	−0.0790 (16)
F1	0.145 (2)	0.0774 (14)	0.152 (2)	−0.0628 (14)	−0.0520 (17)	0.0488 (14)
C3	0.0306 (9)	0.0396 (10)	0.0500 (12)	−0.0060 (8)	0.0035 (8)	−0.0149 (9)
B1	0.0325 (8)	0.0274 (9)	0.0286 (9)	0.0009 (7)	0.0014 (7)	−0.0016 (7)
C8	0.0623 (15)	0.0328 (11)	0.0786 (18)	0.0045 (10)	−0.0137 (13)	0.0030 (11)
C7	0.0430 (12)	0.0708 (17)	0.0647 (16)	0.0017 (11)	0.0180 (11)	−0.0043 (13)
C11B	0.0290 (9)	0.0288 (9)	0.0577 (13)	0.0028 (7)	−0.0003 (9)	−0.0139 (9)
C9	0.0447 (13)	0.0465 (13)	0.096 (2)	0.0029 (10)	−0.0240 (13)	−0.0159 (13)
C12B	0.0348 (10)	0.0346 (11)	0.0518 (13)	−0.0008 (8)	0.0049 (9)	−0.0179 (9)
C10	0.0576 (14)	0.0483 (13)	0.0516 (13)	−0.0172 (11)	−0.0002 (11)	−0.0135 (11)
C16B	0.0355 (12)	0.0406 (17)	0.104 (3)	0.0067 (11)	0.0063 (15)	−0.0228 (15)
C14B	0.0422 (14)	0.0529 (17)	0.084 (2)	0.0033 (12)	−0.0143 (15)	−0.0350 (16)
C13B	0.0582 (16)	0.0333 (12)	0.0767 (17)	0.0001 (11)	0.0128 (13)	0.0028 (11)
C15B	0.091 (2)	0.0653 (18)	0.0448 (12)	−0.0116 (16)	0.0158 (13)	−0.0189 (11)
O4A	0.032 (3)	0.046 (5)	0.055 (8)	0.005 (3)	−0.001 (4)	−0.025 (6)
O5A	0.032 (3)	0.032 (5)	0.073 (12)	0.003 (3)	−0.005 (4)	−0.017 (6)
C12A	0.043 (5)	0.029 (4)	0.063 (8)	0.003 (4)	−0.003 (5)	−0.012 (5)
C11A	0.046 (5)	0.050 (5)	0.056 (7)	0.010 (4)	−0.006 (5)	−0.030 (5)
C14A	0.074 (12)	0.074 (12)	0.053 (7)	0.030 (10)	−0.003 (7)	−0.026 (7)
C16A	0.090 (13)	0.040 (8)	0.069 (10)	0.009 (9)	0.003 (8)	−0.004 (8)
C15A	0.046 (8)	0.032 (10)	0.075 (11)	0.004 (7)	0.002 (9)	−0.015 (7)
C13A	0.038 (8)	0.044 (7)	0.097 (18)	0.015 (6)	−0.006 (8)	−0.035 (8)

### 4-(4,4,5,5-Tetramethyl-1,3,2-dioxaborolan-2-yl)-2-(trimethylsilyl)phenyl trifluoromethanesulfonate (1b) Geometric parameters (Å, º)

**Table:**

S1—O1	1.5620 (16)	C9—H9A	0.9800
S1—O3	1.412 (2)	C9—H9B	0.9800
S1—O2	1.409 (2)	C9—H9C	0.9800
S1—C10	1.830 (2)	C12B—C16B	1.515 (4)
Si1—C1	1.9080 (18)	C12B—C15B	1.527 (4)
Si1—C8	1.865 (2)	C16B—H16A	0.9800
Si1—C7	1.853 (3)	C16B—H16B	0.9800
Si1—C9	1.864 (2)	C16B—H16C	0.9800
O5B—B1	1.373 (3)	C14B—H14A	0.9800
O5B—C12B	1.468 (3)	C14B—H14B	0.9800
O4B—B1	1.368 (3)	C14B—H14C	0.9800
O4B—C11B	1.475 (3)	C13B—H13A	0.9800
O1—C2	1.443 (2)	C13B—H13B	0.9800
F2—C10	1.305 (3)	C13B—H13C	0.9800
F3—C10	1.298 (3)	C15B—H15A	0.9800
C6—H6	0.9500	C15B—H15B	0.9800
C6—C5	1.400 (2)	C15B—H15C	0.9800
C6—C1	1.404 (2)	O4A—C11A	1.42 (2)
C5—C4	1.394 (3)	O5A—C12A	1.43 (2)
C5—B1	1.559 (3)	C12A—C11A	1.57 (2)
C1—C2	1.380 (3)	C12A—C16A	1.51 (3)
C2—C3	1.382 (3)	C12A—C15A	1.46 (3)
C4—H4	0.9500	C11A—C14A	1.51 (3)
C4—C3	1.384 (3)	C11A—C13A	1.45 (3)
F1—C10	1.290 (3)	C14A—H14D	0.9800
C3—H3	0.9500	C14A—H14E	0.9800
B1—O4A	1.26 (2)	C14A—H14F	0.9800
B1—O5A	1.32 (2)	C16A—H16D	0.9800
C8—H8A	0.9800	C16A—H16E	0.9800
C8—H8B	0.9800	C16A—H16F	0.9800
C8—H8C	0.9800	C15A—H15D	0.9800
C7—H7A	0.9800	C15A—H15E	0.9800
C7—H7B	0.9800	C15A—H15F	0.9800
C7—H7C	0.9800	C13A—H13D	0.9800
C11B—C12B	1.555 (3)	C13A—H13E	0.9800
C11B—C14B	1.526 (4)	C13A—H13F	0.9800
C11B—C13B	1.514 (4)		
			
O1—S1—C10	99.77 (10)	F2—C10—S1	109.14 (18)
O3—S1—O1	111.79 (10)	F3—C10—S1	111.62 (16)
O3—S1—C10	107.08 (13)	F3—C10—F2	107.7 (2)
O2—S1—O1	107.72 (13)	F1—C10—S1	110.88 (19)
O2—S1—O3	122.51 (16)	F1—C10—F2	108.9 (2)
O2—S1—C10	105.37 (13)	F1—C10—F3	108.5 (3)
C8—Si1—C1	109.67 (10)	C12B—C16B—H16A	109.5
C7—Si1—C1	108.67 (10)	C12B—C16B—H16B	109.5
C7—Si1—C8	110.84 (13)	C12B—C16B—H16C	109.5
C7—Si1—C9	110.30 (14)	H16A—C16B—H16B	109.5
C9—Si1—C1	107.92 (10)	H16A—C16B—H16C	109.5
C9—Si1—C8	109.38 (13)	H16B—C16B—H16C	109.5
B1—O5B—C12B	105.62 (19)	C11B—C14B—H14A	109.5
B1—O4B—C11B	105.48 (18)	C11B—C14B—H14B	109.5
C2—O1—S1	122.48 (13)	C11B—C14B—H14C	109.5
C5—C6—H6	118.4	H14A—C14B—H14B	109.5
C5—C6—C1	123.12 (16)	H14A—C14B—H14C	109.5
C1—C6—H6	118.4	H14B—C14B—H14C	109.5
C6—C5—B1	121.51 (16)	C11B—C13B—H13A	109.5
C4—C5—C6	118.29 (16)	C11B—C13B—H13B	109.5
C4—C5—B1	120.20 (16)	C11B—C13B—H13C	109.5
C6—C1—Si1	120.92 (13)	H13A—C13B—H13B	109.5
C2—C1—Si1	124.37 (13)	H13A—C13B—H13C	109.5
C2—C1—C6	114.70 (16)	H13B—C13B—H13C	109.5
C1—C2—O1	116.45 (16)	C12B—C15B—H15A	109.5
C1—C2—C3	125.07 (17)	C12B—C15B—H15B	109.5
C3—C2—O1	118.37 (16)	C12B—C15B—H15C	109.5
C5—C4—H4	119.6	H15A—C15B—H15B	109.5
C3—C4—C5	120.70 (17)	H15A—C15B—H15C	109.5
C3—C4—H4	119.6	H15B—C15B—H15C	109.5
C2—C3—C4	118.10 (17)	B1—O4A—C11A	116.4 (16)
C2—C3—H3	120.9	B1—O5A—C12A	113.9 (17)
C4—C3—H3	120.9	O5A—C12A—C11A	98.2 (15)
O5B—B1—C5	121.55 (18)	O5A—C12A—C16A	107.2 (19)
O4B—B1—O5B	114.60 (19)	O5A—C12A—C15A	108 (2)
O4B—B1—C5	123.84 (17)	C16A—C12A—C11A	119.3 (19)
O4A—B1—C5	127.7 (9)	C15A—C12A—C11A	109.3 (18)
O4A—B1—O5A	106.0 (13)	C15A—C12A—C16A	113.0 (17)
O5A—B1—C5	126.2 (9)	O4A—C11A—C12A	98.2 (15)
Si1—C8—H8A	109.5	O4A—C11A—C14A	103.5 (19)
Si1—C8—H8B	109.5	O4A—C11A—C13A	110 (2)
Si1—C8—H8C	109.5	C14A—C11A—C12A	118.1 (19)
H8A—C8—H8B	109.5	C13A—C11A—C12A	110.6 (19)
H8A—C8—H8C	109.5	C13A—C11A—C14A	114.7 (18)
H8B—C8—H8C	109.5	C11A—C14A—H14D	109.5
Si1—C7—H7A	109.5	C11A—C14A—H14E	109.5
Si1—C7—H7B	109.5	C11A—C14A—H14F	109.5
Si1—C7—H7C	109.5	H14D—C14A—H14E	109.5
H7A—C7—H7B	109.5	H14D—C14A—H14F	109.5
H7A—C7—H7C	109.5	H14E—C14A—H14F	109.5
H7B—C7—H7C	109.5	C12A—C16A—H16D	109.5
O4B—C11B—C12B	102.31 (17)	C12A—C16A—H16E	109.5
O4B—C11B—C14B	108.6 (2)	C12A—C16A—H16F	109.5
O4B—C11B—C13B	106.5 (2)	H16D—C16A—H16E	109.5
C14B—C11B—C12B	114.7 (3)	H16D—C16A—H16F	109.5
C13B—C11B—C12B	112.8 (2)	H16E—C16A—H16F	109.5
C13B—C11B—C14B	111.1 (2)	C12A—C15A—H15D	109.5
Si1—C9—H9A	109.5	C12A—C15A—H15E	109.5
Si1—C9—H9B	109.5	C12A—C15A—H15F	109.5
Si1—C9—H9C	109.5	H15D—C15A—H15E	109.5
H9A—C9—H9B	109.5	H15D—C15A—H15F	109.5
H9A—C9—H9C	109.5	H15E—C15A—H15F	109.5
H9B—C9—H9C	109.5	C11A—C13A—H13D	109.5
O5B—C12B—C11B	102.02 (18)	C11A—C13A—H13E	109.5
O5B—C12B—C16B	109.5 (2)	C11A—C13A—H13F	109.5
O5B—C12B—C15B	105.8 (2)	H13D—C13A—H13E	109.5
C16B—C12B—C11B	114.9 (2)	H13D—C13A—H13F	109.5
C16B—C12B—C15B	110.9 (2)	H13E—C13A—H13F	109.5
C15B—C12B—C11B	112.9 (2)		
			
S1—O1—C2—C1	113.56 (17)	O2—S1—C10—F1	−51.3 (2)
S1—O1—C2—C3	−70.1 (2)	B1—O5B—C12B—C11B	−26.2 (3)
Si1—C1—C2—O1	−2.0 (2)	B1—O5B—C12B—C16B	−148.4 (3)
Si1—C1—C2—C3	−178.15 (16)	B1—O5B—C12B—C15B	92.0 (3)
O4B—C11B—C12B—O5B	31.0 (3)	B1—O4B—C11B—C12B	−24.8 (3)
O4B—C11B—C12B—C16B	149.4 (3)	B1—O4B—C11B—C14B	−146.5 (3)
O4B—C11B—C12B—C15B	−82.0 (2)	B1—O4B—C11B—C13B	93.8 (2)
O1—S1—C10—F2	−179.84 (18)	B1—C5—C4—C3	179.85 (18)
O1—S1—C10—F3	−60.9 (2)	B1—O4A—C11A—C12A	22 (3)
O1—S1—C10—F1	60.2 (2)	B1—O4A—C11A—C14A	−100 (2)
O1—C2—C3—C4	−176.13 (18)	B1—O4A—C11A—C13A	137 (2)
O3—S1—O1—C2	−1.04 (19)	B1—O5A—C12A—C11A	24 (3)
O3—S1—C10—F2	−63.3 (2)	B1—O5A—C12A—C16A	−100 (2)
O3—S1—C10—F3	55.7 (2)	B1—O5A—C12A—C15A	137 (2)
O3—S1—C10—F1	176.8 (2)	C11B—O4B—B1—O5B	9.3 (3)
C6—C5—C4—C3	0.5 (3)	C11B—O4B—B1—C5	−170.63 (19)
C6—C5—B1—O5B	168.2 (2)	C12B—O5B—B1—O4B	11.8 (3)
C6—C5—B1—O4B	−11.9 (3)	C12B—O5B—B1—C5	−168.3 (2)
C6—C5—B1—O4A	9.0 (16)	C10—S1—O1—C2	111.90 (16)
C6—C5—B1—O5A	−174.2 (17)	C14B—C11B—C12B—O5B	148.4 (3)
C6—C1—C2—O1	176.92 (16)	C14B—C11B—C12B—C16B	−93.2 (4)
C6—C1—C2—C3	0.8 (3)	C14B—C11B—C12B—C15B	35.4 (3)
C5—C6—C1—Si1	178.10 (13)	C13B—C11B—C12B—O5B	−83.0 (2)
C5—C6—C1—C2	−0.9 (3)	C13B—C11B—C12B—C16B	35.3 (3)
C5—C4—C3—C2	−0.6 (3)	C13B—C11B—C12B—C15B	163.9 (2)
C5—B1—O4A—C11A	169.2 (14)	O4A—B1—O5A—C12A	−12 (3)
C5—B1—O5A—C12A	170.6 (14)	O5A—B1—O4A—C11A	−8 (3)
C1—C6—C5—C4	0.3 (3)	O5A—C12A—C11A—O4A	−24 (2)
C1—C6—C5—B1	−179.04 (16)	O5A—C12A—C11A—C14A	86 (2)
C1—C2—C3—C4	−0.1 (3)	O5A—C12A—C11A—C13A	−139 (2)
C4—C5—B1—O5B	−11.1 (3)	C16A—C12A—C11A—O4A	91 (2)
C4—C5—B1—O4B	168.8 (2)	C16A—C12A—C11A—C14A	−159 (2)
C4—C5—B1—O4A	−170.3 (16)	C16A—C12A—C11A—C13A	−24 (3)
C4—C5—B1—O5A	6.5 (17)	C15A—C12A—C11A—O4A	−137 (2)
O2—S1—O1—C2	−138.38 (17)	C15A—C12A—C11A—C14A	−27 (3)
O2—S1—C10—F2	68.6 (2)	C15A—C12A—C11A—C13A	108 (3)
O2—S1—C10—F3	−172.4 (2)		

### 2-Methyl-4-(4,4,5,5-tetramethyl-1,3,2-dioxaborolan-2-yl)-6-(trimethylsilyl)phenyl trifluoromethanesulfonate (2) Crystal data

**Table:**

C_17_H_26_BF_3_O_5_SSi	*F*(000) = 1840
*M**_r_* = 438.34	*D*_x_ = 1.326 Mg m^−^^3^
Monoclinic, *P*2_1_/*n*	Mo *K*α radiation, λ = 0.71073 Å
*a* = 10.2407 (8) Å	Cell parameters from 9873 reflections
*b* = 12.6295 (10) Å	θ = 2.6–24.6°
*c* = 34.127 (3) Å	µ = 0.25 mm^−^^1^
β = 95.689 (2)°	*T* = 150 K
*V* = 4392.1 (6) Å^3^	Block, clear colourless
*Z* = 8	0.32 × 0.15 × 0.1 mm

### 2-Methyl-4-(4,4,5,5-tetramethyl-1,3,2-dioxaborolan-2-yl)-6-(trimethylsilyl)phenyl trifluoromethanesulfonate (2) Data collection

**Table:**

Bruker APEX-II CCD diffractometer	6597 reflections with *I* > 2σ(*I*)
φ and ω scans	*R*_int_ = 0.091
Absorption correction: multi-scan (SADABS; Krause *et al.*, 2015)	θ_max_ = 26.5°, θ_min_ = 1.7°
*T*_min_ = 0.639, *T*_max_ = 0.745	*h* = −12→12
104215 measured reflections	*k* = −15→15
9044 independent reflections	*l* = −42→42

### 2-Methyl-4-(4,4,5,5-tetramethyl-1,3,2-dioxaborolan-2-yl)-6-(trimethylsilyl)phenyl trifluoromethanesulfonate (2) Refinement

**Table:**

Refinement on *F*^2^	Primary atom site location: structure-invariant direct methods
Least-squares matrix: full	Secondary atom site location: difference Fourier map
*R*[*F*^2^ > 2σ(*F*^2^)] = 0.072	Hydrogen site location: inferred from neighbouring sites
*wR*(*F*^2^) = 0.186	H-atom parameters constrained
*S* = 1.14	*w* = 1/[σ^2^(*F*_o_^2^) + (0.0509*P*)^2^ + 12.3091*P*] where *P* = (*F*_o_^2^ + 2*F*_c_^2^)/3
9044 reflections	(Δ/σ)_max_ = 0.001
521 parameters	Δρ_max_ = 0.60 e Å^−^^3^
0 restraints	Δρ_min_ = −0.48 e Å^−^^3^

### 2-Methyl-4-(4,4,5,5-tetramethyl-1,3,2-dioxaborolan-2-yl)-6-(trimethylsilyl)phenyl trifluoromethanesulfonate (2) Special details

**Table:**

Geometry. All esds (except the esd in the dihedral angle between two l.s. planes) are estimated using the full covariance matrix. The cell esds are taken into account individually in the estimation of esds in distances, angles and torsion angles; correlations between esds in cell parameters are only used when they are defined by crystal symmetry. An approximate (isotropic) treatment of cell esds is used for estimating esds involving l.s. planes.

### 2-Methyl-4-(4,4,5,5-tetramethyl-1,3,2-dioxaborolan-2-yl)-6-(trimethylsilyl)phenyl trifluoromethanesulfonate (2) Fractional atomic coordinates and isotropic or equivalent isotropic displacement parameters (Å^2^)

**Table:**

	*x*	*y*	*z*	*U*_iso_*/*U*_eq_	
S1A	0.39123 (11)	0.84786 (9)	0.43431 (3)	0.0367 (3)	
Si1B	0.75607 (11)	0.94084 (8)	0.41946 (3)	0.0267 (2)	
Si1A	−0.26207 (12)	0.68943 (8)	0.58381 (3)	0.0286 (3)	
S1B	0.07148 (12)	0.59691 (10)	0.55902 (3)	0.0414 (3)	
O5A	−0.1189 (2)	0.4650 (2)	0.75825 (7)	0.0225 (5)	
O4B	0.6223 (2)	0.6987 (2)	0.24683 (7)	0.0240 (6)	
O4A	−0.2966 (2)	0.5687 (2)	0.73914 (7)	0.0233 (6)	
O5B	0.7967 (2)	0.8073 (2)	0.26404 (7)	0.0254 (6)	
O1B	0.5185 (3)	0.7790 (2)	0.43092 (7)	0.0296 (6)	
O1A	−0.0337 (3)	0.5168 (2)	0.57101 (7)	0.0296 (6)	
F3A	0.2121 (3)	0.4247 (3)	0.55218 (9)	0.0686 (10)	
F2B	0.2221 (3)	0.8207 (3)	0.48361 (10)	0.0727 (10)	
O2B	0.3001 (3)	0.8361 (3)	0.40083 (9)	0.0530 (9)	
F1A	0.2615 (3)	0.5144 (3)	0.60534 (9)	0.0771 (11)	
O3A	0.0498 (3)	0.6103 (3)	0.51792 (9)	0.0550 (9)	
C1B	0.6656 (4)	0.8416 (3)	0.38444 (10)	0.0215 (7)	
F3B	0.3002 (5)	0.6761 (3)	0.46304 (13)	0.1001 (15)	
O2A	0.0867 (4)	0.6828 (3)	0.58530 (11)	0.0684 (12)	
C5B	0.6530 (3)	0.7577 (3)	0.31891 (10)	0.0207 (7)	
C13A	−0.1943 (3)	0.4711 (3)	0.79228 (10)	0.0212 (7)	
C5A	−0.1518 (3)	0.5148 (3)	0.68522 (10)	0.0210 (7)	
C2B	0.5681 (4)	0.7713 (3)	0.39265 (10)	0.0233 (8)	
C6B	0.7041 (3)	0.8326 (3)	0.34623 (10)	0.0208 (7)	
H6B	0.768715	0.880265	0.338574	0.025*	
C2A	−0.0768 (4)	0.5180 (3)	0.61022 (10)	0.0240 (8)	
F1B	0.4153 (4)	0.7733 (4)	0.50509 (9)	0.1033 (16)	
C6A	−0.2007 (3)	0.5909 (3)	0.65788 (10)	0.0205 (7)	
H6A	−0.259931	0.642493	0.665967	0.025*	
C12A	−0.2839 (4)	0.5688 (3)	0.78219 (10)	0.0224 (8)	
C1A	−0.1667 (4)	0.5949 (3)	0.61922 (10)	0.0219 (7)	
F2A	0.3151 (3)	0.5730 (4)	0.55004 (11)	0.0955 (14)	
C3B	0.5177 (4)	0.6894 (3)	0.36839 (11)	0.0247 (8)	
C12B	0.6972 (3)	0.7018 (3)	0.21252 (10)	0.0224 (8)	
O3B	0.4257 (4)	0.9493 (3)	0.44926 (13)	0.0666 (11)	
C13B	0.7845 (4)	0.8013 (3)	0.22089 (10)	0.0250 (8)	
C4A	−0.0700 (4)	0.4361 (3)	0.67235 (11)	0.0276 (8)	
H4A	−0.038871	0.381930	0.690204	0.033*	
C4B	0.5622 (4)	0.6855 (3)	0.33111 (11)	0.0245 (8)	
H4B	0.529467	0.631591	0.313347	0.029*	
C16A	−0.1012 (4)	0.4827 (3)	0.82908 (11)	0.0284 (8)	
H16D	−0.045453	0.544942	0.826640	0.043*	
H16E	−0.046157	0.419292	0.832678	0.043*	
H16F	−0.151450	0.491427	0.851859	0.043*	
C3A	−0.0328 (4)	0.4346 (3)	0.63432 (11)	0.0292 (9)	
C14A	−0.2205 (5)	0.6734 (3)	0.79484 (12)	0.0364 (10)	
H14D	−0.207472	0.676600	0.823660	0.055*	
H14E	−0.277579	0.731807	0.784922	0.055*	
H14F	−0.135509	0.679533	0.784134	0.055*	
C17A	−0.2701 (4)	0.3675 (3)	0.79309 (11)	0.0297 (9)	
H17D	−0.316021	0.364708	0.816928	0.045*	
H17E	−0.208849	0.307853	0.793056	0.045*	
H17F	−0.334057	0.363242	0.769811	0.045*	
C15B	0.6026 (4)	0.7080 (4)	0.17555 (11)	0.0346 (10)	
H15A	0.552820	0.641752	0.172286	0.052*	
H15B	0.651893	0.718914	0.152686	0.052*	
H15C	0.541996	0.767193	0.177753	0.052*	
C14B	0.7745 (4)	0.5988 (3)	0.21287 (12)	0.0331 (9)	
H14A	0.836904	0.595992	0.236556	0.050*	
H14B	0.822432	0.595360	0.189432	0.050*	
H14C	0.713967	0.538689	0.212855	0.050*	
B1A	−0.1884 (4)	0.5160 (3)	0.72831 (12)	0.0213 (8)	
C15A	−0.4193 (4)	0.5606 (4)	0.79631 (12)	0.0370 (10)	
H15D	−0.466931	0.501410	0.782941	0.056*	
H15E	−0.467563	0.626539	0.790314	0.056*	
H15F	−0.410897	0.548412	0.824814	0.056*	
C16B	0.9199 (4)	0.7920 (4)	0.20710 (13)	0.0406 (11)	
H16A	0.968909	0.857540	0.213307	0.061*	
H16B	0.912006	0.780209	0.178580	0.061*	
H16C	0.966458	0.732355	0.220461	0.061*	
C11B	0.4230 (4)	0.6088 (4)	0.38077 (13)	0.0386 (10)	
H11A	0.447316	0.589129	0.408303	0.058*	
H11B	0.425377	0.545873	0.364064	0.058*	
H11C	0.334233	0.638668	0.378056	0.058*	
C7A	−0.4221 (5)	0.7105 (4)	0.60373 (13)	0.0427 (11)	
H7AA	−0.478391	0.754610	0.585460	0.064*	
H7AB	−0.407857	0.745982	0.629335	0.064*	
H7AC	−0.464689	0.641948	0.606938	0.064*	
B1B	0.6925 (4)	0.7542 (3)	0.27600 (12)	0.0223 (8)	
C9B	0.9271 (4)	0.9507 (4)	0.40559 (15)	0.0433 (11)	
H9BA	0.976902	1.000912	0.423136	0.065*	
H9BB	0.925356	0.975400	0.378325	0.065*	
H9BC	0.969009	0.880915	0.408021	0.065*	
C17B	0.7191 (5)	0.9031 (4)	0.20578 (14)	0.0457 (12)	
H17A	0.632450	0.908940	0.215451	0.069*	
H17B	0.709381	0.902582	0.176925	0.069*	
H17C	0.773322	0.963653	0.215210	0.069*	
C9A	−0.2931 (5)	0.6271 (4)	0.53447 (12)	0.0480 (13)	
H9AA	−0.210618	0.622567	0.522252	0.072*	
H9AB	−0.356223	0.670045	0.517869	0.072*	
H9AC	−0.328818	0.555816	0.537259	0.072*	
C8B	0.6781 (5)	1.0733 (3)	0.41309 (14)	0.0440 (11)	
H8BA	0.589912	1.070778	0.421872	0.066*	
H8BB	0.672056	1.093520	0.385245	0.066*	
H8BC	0.731247	1.125530	0.428794	0.066*	
C7B	0.7648 (5)	0.8966 (4)	0.47155 (12)	0.0494 (13)	
H7BA	0.676286	0.895644	0.480174	0.074*	
H7BB	0.820087	0.945592	0.488139	0.074*	
H7BC	0.802467	0.825317	0.473826	0.074*	
C11A	0.0460 (5)	0.3433 (4)	0.62091 (14)	0.0487 (13)	
H11D	0.139683	0.357339	0.627585	0.073*	
H11E	0.028084	0.334841	0.592340	0.073*	
H11F	0.021552	0.278339	0.634074	0.073*	
C10B	0.3294 (6)	0.7731 (5)	0.47419 (15)	0.0565 (15)	
C10A	0.2237 (5)	0.5213 (6)	0.56719 (14)	0.0584 (15)	
C8A	−0.1799 (6)	0.8207 (4)	0.58119 (16)	0.0554 (14)	
H8AA	−0.152570	0.845925	0.607900	0.083*	
H8AB	−0.241322	0.871522	0.567751	0.083*	
H8AC	−0.102792	0.813594	0.566545	0.083*	

### 2-Methyl-4-(4,4,5,5-tetramethyl-1,3,2-dioxaborolan-2-yl)-6-(trimethylsilyl)phenyl trifluoromethanesulfonate (2) Atomic displacement parameters (Å^2^)

**Table:**

	*U*^11^	*U*^22^	*U*^33^	*U*^12^	*U*^13^	*U*^23^
S1A	0.0388 (6)	0.0388 (6)	0.0350 (5)	0.0072 (5)	0.0163 (5)	0.0046 (5)
Si1B	0.0365 (6)	0.0190 (5)	0.0245 (5)	−0.0014 (5)	0.0031 (4)	−0.0014 (4)
Si1A	0.0444 (7)	0.0194 (5)	0.0218 (5)	0.0039 (5)	0.0025 (5)	0.0018 (4)
S1B	0.0440 (7)	0.0485 (7)	0.0347 (6)	−0.0136 (5)	0.0183 (5)	−0.0067 (5)
O5A	0.0180 (12)	0.0278 (14)	0.0225 (12)	0.0041 (11)	0.0061 (10)	0.0033 (10)
O4B	0.0189 (13)	0.0299 (15)	0.0243 (13)	−0.0052 (11)	0.0076 (10)	−0.0023 (11)
O4A	0.0212 (13)	0.0264 (14)	0.0223 (12)	0.0057 (11)	0.0028 (10)	0.0044 (10)
O5B	0.0213 (13)	0.0312 (15)	0.0243 (13)	−0.0085 (11)	0.0064 (10)	−0.0052 (11)
O1B	0.0372 (16)	0.0307 (15)	0.0221 (13)	0.0006 (12)	0.0091 (11)	0.0028 (11)
O1A	0.0351 (15)	0.0314 (15)	0.0242 (13)	−0.0021 (12)	0.0118 (11)	−0.0039 (11)
F3A	0.060 (2)	0.098 (3)	0.0511 (18)	0.0208 (19)	0.0204 (15)	−0.0170 (18)
F2B	0.062 (2)	0.092 (3)	0.072 (2)	0.0189 (19)	0.0448 (17)	0.0151 (19)
O2B	0.0451 (19)	0.077 (3)	0.0378 (17)	0.0178 (18)	0.0069 (15)	0.0078 (17)
F1A	0.057 (2)	0.132 (3)	0.0409 (17)	−0.010 (2)	−0.0042 (14)	−0.0058 (19)
O3A	0.055 (2)	0.070 (3)	0.0422 (18)	−0.0147 (19)	0.0159 (16)	0.0111 (17)
C1B	0.0261 (19)	0.0139 (17)	0.0248 (18)	0.0034 (14)	0.0036 (14)	0.0010 (14)
F3B	0.141 (4)	0.055 (2)	0.121 (3)	−0.011 (2)	0.094 (3)	0.012 (2)
O2A	0.084 (3)	0.057 (2)	0.071 (2)	−0.040 (2)	0.043 (2)	−0.028 (2)
C5B	0.0181 (17)	0.0198 (18)	0.0244 (18)	0.0042 (14)	0.0035 (14)	0.0003 (14)
C13A	0.0189 (17)	0.0250 (19)	0.0203 (17)	0.0020 (15)	0.0051 (14)	0.0023 (14)
C5A	0.0178 (17)	0.0213 (19)	0.0239 (18)	−0.0034 (14)	0.0020 (14)	−0.0001 (14)
C2B	0.0255 (19)	0.0238 (19)	0.0216 (17)	0.0027 (15)	0.0079 (14)	0.0042 (15)
C6B	0.0226 (18)	0.0118 (17)	0.0283 (18)	0.0013 (14)	0.0043 (14)	0.0019 (14)
C2A	0.0274 (19)	0.026 (2)	0.0198 (17)	−0.0034 (16)	0.0065 (14)	−0.0040 (15)
F1B	0.100 (3)	0.175 (5)	0.0381 (18)	0.052 (3)	0.0207 (18)	0.033 (2)
C6A	0.0247 (18)	0.0142 (17)	0.0230 (17)	−0.0007 (14)	0.0036 (14)	−0.0020 (13)
C12A	0.0232 (18)	0.0233 (19)	0.0212 (17)	0.0081 (15)	0.0049 (14)	0.0028 (14)
C1A	0.0290 (19)	0.0151 (18)	0.0217 (17)	−0.0052 (15)	0.0035 (14)	−0.0024 (14)
F2A	0.0431 (18)	0.169 (4)	0.079 (2)	−0.022 (2)	0.0286 (17)	0.011 (3)
C3B	0.0223 (18)	0.0230 (19)	0.0289 (19)	−0.0018 (15)	0.0039 (15)	0.0031 (15)
C12B	0.0185 (17)	0.029 (2)	0.0206 (17)	−0.0018 (15)	0.0065 (14)	−0.0013 (15)
O3B	0.061 (2)	0.042 (2)	0.100 (3)	0.0050 (18)	0.025 (2)	−0.017 (2)
C13B	0.0248 (19)	0.027 (2)	0.0238 (18)	−0.0029 (16)	0.0074 (15)	−0.0013 (15)
C4A	0.030 (2)	0.026 (2)	0.0266 (19)	0.0055 (17)	0.0013 (16)	0.0030 (16)
C4B	0.028 (2)	0.0200 (19)	0.0250 (18)	−0.0034 (15)	0.0024 (15)	−0.0024 (15)
C16A	0.0234 (19)	0.036 (2)	0.0255 (19)	0.0010 (17)	0.0011 (15)	0.0027 (17)
C3A	0.030 (2)	0.028 (2)	0.030 (2)	0.0079 (17)	0.0049 (16)	−0.0034 (16)
C14A	0.053 (3)	0.024 (2)	0.033 (2)	0.0017 (19)	0.0073 (19)	−0.0020 (17)
C17A	0.034 (2)	0.024 (2)	0.031 (2)	−0.0022 (17)	0.0066 (17)	0.0042 (16)
C15B	0.032 (2)	0.046 (3)	0.026 (2)	−0.0036 (19)	0.0019 (17)	0.0002 (18)
C14B	0.036 (2)	0.027 (2)	0.038 (2)	0.0013 (18)	0.0102 (18)	−0.0036 (18)
B1A	0.0174 (19)	0.020 (2)	0.026 (2)	−0.0034 (16)	0.0007 (16)	0.0007 (16)
C15A	0.028 (2)	0.050 (3)	0.036 (2)	0.016 (2)	0.0134 (17)	0.011 (2)
C16B	0.033 (2)	0.052 (3)	0.039 (2)	−0.018 (2)	0.0168 (19)	−0.012 (2)
C11B	0.043 (3)	0.037 (2)	0.037 (2)	−0.018 (2)	0.0107 (19)	0.0007 (19)
C7A	0.051 (3)	0.040 (3)	0.037 (2)	0.020 (2)	0.002 (2)	0.003 (2)
B1B	0.020 (2)	0.021 (2)	0.027 (2)	0.0035 (16)	0.0030 (16)	0.0004 (17)
C9B	0.033 (2)	0.036 (3)	0.061 (3)	−0.010 (2)	0.003 (2)	−0.014 (2)
C17B	0.069 (3)	0.029 (2)	0.041 (3)	0.001 (2)	0.013 (2)	0.003 (2)
C9A	0.063 (3)	0.054 (3)	0.025 (2)	0.017 (3)	−0.007 (2)	−0.006 (2)
C8B	0.061 (3)	0.024 (2)	0.046 (3)	0.004 (2)	0.003 (2)	−0.0076 (19)
C7B	0.070 (3)	0.047 (3)	0.030 (2)	−0.014 (3)	−0.004 (2)	0.003 (2)
C11A	0.062 (3)	0.042 (3)	0.045 (3)	0.025 (2)	0.015 (2)	0.002 (2)
C10B	0.058 (3)	0.074 (4)	0.042 (3)	0.022 (3)	0.028 (2)	0.017 (3)
C10A	0.038 (3)	0.102 (5)	0.036 (3)	−0.013 (3)	0.009 (2)	−0.005 (3)
C8A	0.082 (4)	0.028 (3)	0.056 (3)	−0.005 (3)	0.004 (3)	0.014 (2)

### 2-Methyl-4-(4,4,5,5-tetramethyl-1,3,2-dioxaborolan-2-yl)-6-(trimethylsilyl)phenyl trifluoromethanesulfonate (2) Geometric parameters (Å, º)

**Table:**

S1A—O1B	1.581 (3)	C12B—C14B	1.522 (5)
S1A—O2B	1.409 (3)	C13B—C16B	1.512 (5)
S1A—O3B	1.411 (4)	C13B—C17B	1.517 (6)
S1A—C10B	1.821 (5)	C4A—H4A	0.9500
Si1B—C1B	1.908 (4)	C4A—C3A	1.388 (5)
Si1B—C9B	1.863 (5)	C4B—H4B	0.9500
Si1B—C8B	1.857 (5)	C16A—H16D	0.9800
Si1B—C7B	1.857 (4)	C16A—H16E	0.9800
Si1A—C1A	1.899 (4)	C16A—H16F	0.9800
Si1A—C7A	1.855 (5)	C3A—C11A	1.505 (6)
Si1A—C9A	1.857 (4)	C14A—H14D	0.9800
Si1A—C8A	1.865 (5)	C14A—H14E	0.9800
S1B—O1A	1.561 (3)	C14A—H14F	0.9800
S1B—O3A	1.408 (3)	C17A—H17D	0.9800
S1B—O2A	1.406 (4)	C17A—H17E	0.9800
S1B—C10A	1.826 (6)	C17A—H17F	0.9800
O5A—C13A	1.459 (4)	C15B—H15A	0.9800
O5A—B1A	1.349 (5)	C15B—H15B	0.9800
O4B—C12B	1.463 (4)	C15B—H15C	0.9800
O4B—B1B	1.362 (5)	C14B—H14A	0.9800
O4A—C12A	1.462 (4)	C14B—H14B	0.9800
O4A—B1A	1.374 (5)	C14B—H14C	0.9800
O5B—C13B	1.467 (4)	C15A—H15D	0.9800
O5B—B1B	1.357 (5)	C15A—H15E	0.9800
O1B—C2B	1.451 (4)	C15A—H15F	0.9800
O1A—C2A	1.450 (4)	C16B—H16A	0.9800
F3A—C10A	1.324 (7)	C16B—H16B	0.9800
F2B—C10B	1.320 (6)	C16B—H16C	0.9800
F1A—C10A	1.324 (6)	C11B—H11A	0.9800
C1B—C2B	1.385 (5)	C11B—H11B	0.9800
C1B—C6B	1.404 (5)	C11B—H11C	0.9800
F3B—C10B	1.309 (7)	C7A—H7AA	0.9800
C5B—C6B	1.393 (5)	C7A—H7AB	0.9800
C5B—C4B	1.395 (5)	C7A—H7AC	0.9800
C5B—B1B	1.558 (5)	C9B—H9BA	0.9800
C13A—C12A	1.556 (5)	C9B—H9BB	0.9800
C13A—C16A	1.507 (5)	C9B—H9BC	0.9800
C13A—C17A	1.522 (5)	C17B—H17A	0.9800
C5A—C6A	1.397 (5)	C17B—H17B	0.9800
C5A—C4A	1.398 (5)	C17B—H17C	0.9800
C5A—B1A	1.553 (5)	C9A—H9AA	0.9800
C2B—C3B	1.392 (5)	C9A—H9AB	0.9800
C6B—H6B	0.9500	C9A—H9AC	0.9800
C2A—C1A	1.393 (5)	C8B—H8BA	0.9800
C2A—C3A	1.384 (5)	C8B—H8BB	0.9800
F1B—C10B	1.305 (7)	C8B—H8BC	0.9800
C6A—H6A	0.9500	C7B—H7BA	0.9800
C6A—C1A	1.399 (5)	C7B—H7BB	0.9800
C12A—C14A	1.516 (6)	C7B—H7BC	0.9800
C12A—C15A	1.516 (5)	C11A—H11D	0.9800
F2A—C10A	1.325 (6)	C11A—H11E	0.9800
C3B—C4B	1.394 (5)	C11A—H11F	0.9800
C3B—C11B	1.496 (5)	C8A—H8AA	0.9800
C12B—C13B	1.552 (5)	C8A—H8AB	0.9800
C12B—C15B	1.515 (5)	C8A—H8AC	0.9800
			
O1B—S1A—C10B	96.9 (2)	C13A—C17A—H17D	109.5
O2B—S1A—O1B	111.53 (18)	C13A—C17A—H17E	109.5
O2B—S1A—O3B	120.7 (3)	C13A—C17A—H17F	109.5
O2B—S1A—C10B	107.5 (3)	H17D—C17A—H17E	109.5
O3B—S1A—O1B	110.4 (2)	H17D—C17A—H17F	109.5
O3B—S1A—C10B	107.1 (3)	H17E—C17A—H17F	109.5
C9B—Si1B—C1B	107.15 (18)	C12B—C15B—H15A	109.5
C8B—Si1B—C1B	110.02 (19)	C12B—C15B—H15B	109.5
C8B—Si1B—C9B	108.3 (2)	C12B—C15B—H15C	109.5
C7B—Si1B—C1B	112.11 (19)	H15A—C15B—H15B	109.5
C7B—Si1B—C9B	107.8 (2)	H15A—C15B—H15C	109.5
C7B—Si1B—C8B	111.2 (2)	H15B—C15B—H15C	109.5
C7A—Si1A—C1A	105.92 (18)	C12B—C14B—H14A	109.5
C7A—Si1A—C9A	108.4 (2)	C12B—C14B—H14B	109.5
C7A—Si1A—C8A	108.0 (2)	C12B—C14B—H14C	109.5
C9A—Si1A—C1A	109.97 (19)	H14A—C14B—H14B	109.5
C9A—Si1A—C8A	111.8 (2)	H14A—C14B—H14C	109.5
C8A—Si1A—C1A	112.5 (2)	H14B—C14B—H14C	109.5
O1A—S1B—C10A	102.8 (2)	O5A—B1A—O4A	113.9 (3)
O3A—S1B—O1A	107.10 (18)	O5A—B1A—C5A	123.6 (3)
O3A—S1B—C10A	105.2 (2)	O4A—B1A—C5A	122.4 (3)
O2A—S1B—O1A	111.60 (18)	C12A—C15A—H15D	109.5
O2A—S1B—O3A	122.6 (3)	C12A—C15A—H15E	109.5
O2A—S1B—C10A	105.5 (3)	C12A—C15A—H15F	109.5
B1A—O5A—C13A	107.3 (3)	H15D—C15A—H15E	109.5
B1B—O4B—C12B	106.9 (3)	H15D—C15A—H15F	109.5
B1A—O4A—C12A	106.1 (3)	H15E—C15A—H15F	109.5
B1B—O5B—C13B	106.6 (3)	C13B—C16B—H16A	109.5
C2B—O1B—S1A	117.9 (2)	C13B—C16B—H16B	109.5
C2A—O1A—S1B	121.1 (2)	C13B—C16B—H16C	109.5
C2B—C1B—Si1B	127.5 (3)	H16A—C16B—H16B	109.5
C2B—C1B—C6B	114.3 (3)	H16A—C16B—H16C	109.5
C6B—C1B—Si1B	118.1 (3)	H16B—C16B—H16C	109.5
C6B—C5B—C4B	117.5 (3)	C3B—C11B—H11A	109.5
C6B—C5B—B1B	122.2 (3)	C3B—C11B—H11B	109.5
C4B—C5B—B1B	120.3 (3)	C3B—C11B—H11C	109.5
O5A—C13A—C12A	102.2 (3)	H11A—C11B—H11B	109.5
O5A—C13A—C16A	109.1 (3)	H11A—C11B—H11C	109.5
O5A—C13A—C17A	106.3 (3)	H11B—C11B—H11C	109.5
C16A—C13A—C12A	114.7 (3)	Si1A—C7A—H7AA	109.5
C16A—C13A—C17A	110.5 (3)	Si1A—C7A—H7AB	109.5
C17A—C13A—C12A	113.3 (3)	Si1A—C7A—H7AC	109.5
C6A—C5A—C4A	117.7 (3)	H7AA—C7A—H7AB	109.5
C6A—C5A—B1A	121.4 (3)	H7AA—C7A—H7AC	109.5
C4A—C5A—B1A	120.8 (3)	H7AB—C7A—H7AC	109.5
C1B—C2B—O1B	117.6 (3)	O4B—B1B—C5B	122.3 (3)
C1B—C2B—C3B	126.0 (3)	O5B—B1B—O4B	114.1 (3)
C3B—C2B—O1B	116.3 (3)	O5B—B1B—C5B	123.6 (3)
C1B—C6B—H6B	118.1	Si1B—C9B—H9BA	109.5
C5B—C6B—C1B	123.7 (3)	Si1B—C9B—H9BB	109.5
C5B—C6B—H6B	118.1	Si1B—C9B—H9BC	109.5
C1A—C2A—O1A	118.6 (3)	H9BA—C9B—H9BB	109.5
C3A—C2A—O1A	115.5 (3)	H9BA—C9B—H9BC	109.5
C3A—C2A—C1A	125.7 (3)	H9BB—C9B—H9BC	109.5
C5A—C6A—H6A	118.4	C13B—C17B—H17A	109.5
C5A—C6A—C1A	123.3 (3)	C13B—C17B—H17B	109.5
C1A—C6A—H6A	118.4	C13B—C17B—H17C	109.5
O4A—C12A—C13A	102.3 (3)	H17A—C17B—H17B	109.5
O4A—C12A—C14A	106.3 (3)	H17A—C17B—H17C	109.5
O4A—C12A—C15A	109.1 (3)	H17B—C17B—H17C	109.5
C14A—C12A—C13A	113.7 (3)	Si1A—C9A—H9AA	109.5
C14A—C12A—C15A	110.4 (3)	Si1A—C9A—H9AB	109.5
C15A—C12A—C13A	114.4 (3)	Si1A—C9A—H9AC	109.5
C2A—C1A—Si1A	127.4 (3)	H9AA—C9A—H9AB	109.5
C2A—C1A—C6A	114.5 (3)	H9AA—C9A—H9AC	109.5
C6A—C1A—Si1A	117.6 (3)	H9AB—C9A—H9AC	109.5
C2B—C3B—C4B	115.7 (3)	Si1B—C8B—H8BA	109.5
C2B—C3B—C11B	123.4 (3)	Si1B—C8B—H8BB	109.5
C4B—C3B—C11B	120.9 (3)	Si1B—C8B—H8BC	109.5
O4B—C12B—C13B	102.2 (3)	H8BA—C8B—H8BB	109.5
O4B—C12B—C15B	109.0 (3)	H8BA—C8B—H8BC	109.5
O4B—C12B—C14B	106.5 (3)	H8BB—C8B—H8BC	109.5
C15B—C12B—C13B	114.9 (3)	Si1B—C7B—H7BA	109.5
C15B—C12B—C14B	109.9 (3)	Si1B—C7B—H7BB	109.5
C14B—C12B—C13B	113.6 (3)	Si1B—C7B—H7BC	109.5
O5B—C13B—C12B	102.5 (3)	H7BA—C7B—H7BB	109.5
O5B—C13B—C16B	109.1 (3)	H7BA—C7B—H7BC	109.5
O5B—C13B—C17B	106.7 (3)	H7BB—C7B—H7BC	109.5
C16B—C13B—C12B	114.2 (3)	C3A—C11A—H11D	109.5
C16B—C13B—C17B	110.3 (4)	C3A—C11A—H11E	109.5
C17B—C13B—C12B	113.4 (3)	C3A—C11A—H11F	109.5
C5A—C4A—H4A	118.9	H11D—C11A—H11E	109.5
C3A—C4A—C5A	122.1 (4)	H11D—C11A—H11F	109.5
C3A—C4A—H4A	118.9	H11E—C11A—H11F	109.5
C5B—C4B—H4B	118.8	F2B—C10B—S1A	107.7 (4)
C3B—C4B—C5B	122.5 (3)	F3B—C10B—S1A	110.8 (4)
C3B—C4B—H4B	118.8	F3B—C10B—F2B	109.0 (5)
C13A—C16A—H16D	109.5	F1B—C10B—S1A	110.3 (4)
C13A—C16A—H16E	109.5	F1B—C10B—F2B	108.2 (4)
C13A—C16A—H16F	109.5	F1B—C10B—F3B	110.7 (5)
H16D—C16A—H16E	109.5	F3A—C10A—S1B	112.4 (4)
H16D—C16A—H16F	109.5	F3A—C10A—F1A	109.0 (5)
H16E—C16A—H16F	109.5	F3A—C10A—F2A	108.9 (5)
C2A—C3A—C4A	116.2 (3)	F1A—C10A—S1B	110.3 (4)
C2A—C3A—C11A	123.7 (4)	F1A—C10A—F2A	108.5 (4)
C4A—C3A—C11A	120.0 (4)	F2A—C10A—S1B	107.7 (5)
C12A—C14A—H14D	109.5	Si1A—C8A—H8AA	109.5
C12A—C14A—H14E	109.5	Si1A—C8A—H8AB	109.5
C12A—C14A—H14F	109.5	Si1A—C8A—H8AC	109.5
H14D—C14A—H14E	109.5	H8AA—C8A—H8AB	109.5
H14D—C14A—H14F	109.5	H8AA—C8A—H8AC	109.5
H14E—C14A—H14F	109.5	H8AB—C8A—H8AC	109.5
			
S1A—O1B—C2B—C1B	94.5 (4)	C12A—O4A—B1A—C5A	−169.4 (3)
S1A—O1B—C2B—C3B	−89.0 (4)	C1A—C2A—C3A—C4A	−8.2 (6)
Si1B—C1B—C2B—O1B	6.3 (5)	C1A—C2A—C3A—C11A	169.2 (4)
Si1B—C1B—C2B—C3B	−169.8 (3)	C12B—O4B—B1B—O5B	8.9 (4)
Si1B—C1B—C6B—C5B	174.9 (3)	C12B—O4B—B1B—C5B	−172.6 (3)
S1B—O1A—C2A—C1A	78.2 (4)	O3B—S1A—O1B—C2B	−98.7 (3)
S1B—O1A—C2A—C3A	−107.6 (4)	O3B—S1A—C10B—F2B	63.4 (5)
O5A—C13A—C12A—O4A	27.9 (3)	O3B—S1A—C10B—F3B	−177.5 (4)
O5A—C13A—C12A—C14A	−86.2 (3)	O3B—S1A—C10B—F1B	−54.5 (5)
O5A—C13A—C12A—C15A	145.6 (3)	C13B—O5B—B1B—O4B	9.8 (4)
O4B—C12B—C13B—O5B	27.1 (3)	C13B—O5B—B1B—C5B	−168.7 (3)
O4B—C12B—C13B—C16B	145.0 (3)	C4A—C5A—C6A—C1A	−3.2 (5)
O4B—C12B—C13B—C17B	−87.5 (4)	C4A—C5A—B1A—O5A	19.8 (6)
O1B—S1A—C10B—F2B	177.2 (4)	C4A—C5A—B1A—O4A	−159.3 (4)
O1B—S1A—C10B—F3B	−63.6 (4)	C4B—C5B—C6B—C1B	−2.6 (5)
O1B—S1A—C10B—F1B	59.3 (4)	C4B—C5B—B1B—O4B	14.6 (5)
O1B—C2B—C3B—C4B	177.5 (3)	C4B—C5B—B1B—O5B	−167.1 (4)
O1B—C2B—C3B—C11B	−3.1 (5)	C16A—C13A—C12A—O4A	145.8 (3)
O1A—S1B—C10A—F3A	46.8 (4)	C16A—C13A—C12A—C14A	31.7 (4)
O1A—S1B—C10A—F1A	−75.1 (4)	C16A—C13A—C12A—C15A	−96.4 (4)
O1A—S1B—C10A—F2A	166.7 (4)	C3A—C2A—C1A—Si1A	−163.6 (3)
O1A—C2A—C1A—Si1A	9.8 (5)	C3A—C2A—C1A—C6A	7.6 (6)
O1A—C2A—C1A—C6A	−178.9 (3)	C17A—C13A—C12A—O4A	−86.1 (3)
O1A—C2A—C3A—C4A	178.2 (3)	C17A—C13A—C12A—C14A	159.8 (3)
O1A—C2A—C3A—C11A	−4.4 (6)	C17A—C13A—C12A—C15A	31.7 (4)
O2B—S1A—O1B—C2B	38.3 (3)	C15B—C12B—C13B—O5B	145.0 (3)
O2B—S1A—C10B—F2B	−67.6 (5)	C15B—C12B—C13B—C16B	−97.1 (4)
O2B—S1A—C10B—F3B	51.5 (5)	C15B—C12B—C13B—C17B	30.4 (5)
O2B—S1A—C10B—F1B	174.5 (4)	C14B—C12B—C13B—O5B	−87.3 (3)
O3A—S1B—O1A—C2A	−153.7 (3)	C14B—C12B—C13B—C16B	30.6 (5)
O3A—S1B—C10A—F3A	−65.2 (4)	C14B—C12B—C13B—C17B	158.1 (3)
O3A—S1B—C10A—F1A	172.9 (4)	B1A—O5A—C13A—C12A	−22.2 (4)
O3A—S1B—C10A—F2A	54.7 (4)	B1A—O5A—C13A—C16A	−144.0 (3)
C1B—C2B—C3B—C4B	−6.4 (6)	B1A—O5A—C13A—C17A	96.8 (3)
C1B—C2B—C3B—C11B	173.0 (4)	B1A—O4A—C12A—C13A	−24.1 (3)
O2A—S1B—O1A—C2A	−16.9 (4)	B1A—O4A—C12A—C14A	95.4 (3)
O2A—S1B—C10A—F3A	163.8 (3)	B1A—O4A—C12A—C15A	−145.6 (3)
O2A—S1B—C10A—F1A	42.0 (5)	B1A—C5A—C6A—C1A	177.7 (3)
O2A—S1B—C10A—F2A	−76.2 (4)	B1A—C5A—C4A—C3A	−178.2 (4)
C13A—O5A—B1A—O4A	7.8 (4)	C11B—C3B—C4B—C5B	−178.4 (4)
C13A—O5A—B1A—C5A	−171.3 (3)	C7A—Si1A—C1A—C2A	147.1 (3)
C5A—C6A—C1A—Si1A	170.6 (3)	C7A—Si1A—C1A—C6A	−23.9 (3)
C5A—C6A—C1A—C2A	−1.6 (5)	B1B—O4B—C12B—C13B	−22.3 (4)
C5A—C4A—C3A—C2A	2.6 (6)	B1B—O4B—C12B—C15B	−144.3 (3)
C5A—C4A—C3A—C11A	−174.9 (4)	B1B—O4B—C12B—C14B	97.2 (3)
C2B—C1B—C6B—C5B	−2.0 (5)	B1B—O5B—C13B—C12B	−22.8 (4)
C2B—C3B—C4B—C5B	1.0 (6)	B1B—O5B—C13B—C16B	−144.2 (3)
C6B—C1B—C2B—O1B	−177.1 (3)	B1B—O5B—C13B—C17B	96.6 (4)
C6B—C1B—C2B—C3B	6.8 (5)	B1B—C5B—C6B—C1B	176.6 (3)
C6B—C5B—C4B—C3B	3.1 (5)	B1B—C5B—C4B—C3B	−176.0 (3)
C6B—C5B—B1B—O4B	−164.5 (3)	C9A—Si1A—C1A—C2A	30.2 (4)
C6B—C5B—B1B—O5B	13.8 (6)	C9A—Si1A—C1A—C6A	−140.8 (3)
C6A—C5A—C4A—C3A	2.7 (6)	C10B—S1A—O1B—C2B	150.1 (3)
C6A—C5A—B1A—O5A	−161.1 (3)	C10A—S1B—O1A—C2A	95.7 (3)
C6A—C5A—B1A—O4A	19.8 (5)	C8A—Si1A—C1A—C2A	−95.1 (4)
C12A—O4A—B1A—O5A	11.4 (4)	C8A—Si1A—C1A—C6A	93.9 (3)
